# Distinct microbial and metabolic shifts characterize acute coronary syndrome and recovery

**DOI:** 10.1002/imt2.70079

**Published:** 2025-09-19

**Authors:** Jing Xu, Die Dai, Yanan Yang, Shanshan Gao, Jingang Yang, Chaoran Dong, Weixian Yang, Jiansong Yuan, Tianjie Wang, Tao Tian, Yanmin Yang, Fang Luo, Ping Jiang, Chao Wu, Xiaolu Sun, Yonggang Sui, Guofeng Gao, Wentao Ma, Yuan Wu, Jun Zhang, Jia Li, Chao Guo, Cheng Cui, Tingting Guo, Xueyan Zhao, Jinqing Yuan, Shubin Qiao, Fenghuan Hu, Xiaojin Gao, Xiaoliang Luo, Haoran Peng, Daoming Wang, Jiqiu Wu, Chongming Wu, Jiuming He, Wei‐Hua Chen, Yuejin Yang, Jingyuan Fu

**Affiliations:** ^1^ Department of Cardiology State Key Laboratory of Cardiovascular Diseases, Fuwai Hospital, National Center for Cardiovascular Diseases, Chinese Academy of Medical Sciences and Peking Union Medical College Beijing China; ^2^ Department of Genetics & Department of Pediatrics University Medical Center Groningen, University of Groningen Groningen The Netherlands; ^3^ Key Laboratory of Molecular Biophysics of the Ministry of Education, Hubei Key Laboratory of Bioinformatics and Molecular Imaging, Department of Bioinformatics and Systems Biology, Center for Artificial Intelligence Biology, College of Life Science and Technology Huazhong University of Science and Technology Wuhan China; ^4^ School of Chinese Materia Medica Tianjin University of Traditional Chinese Medicine Tianjin China; ^5^ State Key Laboratory of Bioactive Substance and Function of Natural Medicines, Institute of Materia Medica, Chinese Academy of Medical Sciences and Peking Union Medical College Beijing China; ^6^ School of Biological Science Jining Medical University Rizhao China

**Keywords:** acute coronary syndrome, biomarkers, coronary artery disease, gut microbiome, metabolomics

## Abstract

Early identification of patients at risk of acute coronary syndrome (ACS) remains a major unmet need, particularly among those with stable coronary artery disease (sCAD), where timely intervention could markedly improve outcomes. The gut microbiota has been implicated in coronary artery disease (CAD), but its ability to distinguish ACS from sCAD is not well defined. Here, we performed cross‐sectional multi‐omics profiling of fecal microbiota and plasma metabolites in 548 individuals, including participants with normal coronary arteries (*N* = 175), primary sCAD (*N* = 161), and ACS (*N* = 212). To assess whether disease‐associated changes resolve with treatment, we further analyzed an independent cohort of ACS patients (*N* = 52) who transitioned to sCAD following standard therapy. We identified profound ACS‐associated alterations in gut microbial composition and systemic metabolism, marked by enrichment of pro‐inflammatory taxa such as *Streptococcus* spp. and elevated circulating levels of 3‐hydroxybutyrate (3‐HB). Strikingly, many of these ACS‐specific microbial and metabolic signatures, including 3‐HB and related microbial functional pathways, were restored toward sCAD‐like levels after clinical recovery. Integrative models combining microbial taxa, metabolites, and clinical biomarkers robustly discriminated ACS from healthy controls (AUC = 0.91) and from sCAD (AUC = 0.83), significantly outperforming clinical markers alone (AUC = 0.69 for NCA vs. ACS; 0.59 for sCAD vs. ACS). These findings establish the gut microbiome and its metabolic outputs as key discriminators of ACS, reveal their dynamic resolution during disease recovery, and highlight their potential as biomarkers and therapeutic targets for cardiovascular risk stratification and management.

## Highlights

- Gut microbiota and circulating metabolites display stage‐specific alterations across normal coronary arteries, stable coronary artery disease (sCAD), and acute coronary syndrome (ACS), underscoring the gut–heart axis in CAD progression.
- ACS‐specific microbial and metabolic signatures were identified and validated in a recovery cohort, demonstrating partially reversible, stage‐specific shifts.
- Multi‐omics machine learning models accurately stratified CAD subtypes, surpassing the predictive power of clinical risk factors alone.

## Graphical abstract

This study explores the gut–heart axis in coronary artery disease (CAD) using multi‐omics integration of clinical, microbiome and metabolome data. Across healthy controls, stable CAD (sCAD), acute coronary syndrome (ACS), and recovery, we identify stage‐specific microbial dysbiosis and metabolite alterations that mark the transition from stability to acute events. ACS is characterized by pro‐inflammatory taxa (e.g., *Streptococcus* spp.) and elevated metabolites such as 3‐hydroxybutyrate, which normalize following recovery. Multi‐omics models robustly distinguish ACS from sCAD and healthy states, outperforming clinical markers alone. These findings reveal dynamic microbial–metabolic signatures underlying plaque destabilization and resolution, and highlight opportunities for microbiome‐based biomarkers and therapeutic strategies in CAD management.

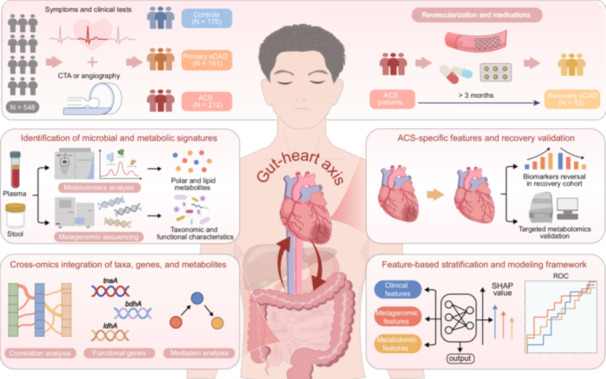

## INTRODUCTION

Coronary artery disease (CAD) remains the leading cause of mortality and morbidity worldwide. Overall cardiovascular disease deaths rose from 12.4 million in 1990 to 19.8 million in 2022, with CAD accounting for the largest proportion [1]. CAD manifests in two primary clinical forms: chronic or stable CAD (sCAD) and acute coronary syndromes (ACS) [2]. ACS, the more fatal subtype, includes sudden cardiac death (SCD), ST‐segment elevated myocardial infarction (STEMI), non‐STEMI (NSTEMI), and unstable angina pectoris, most often resulting from the rupture or erosion of vulnerable atherosclerotic plaques [3].

The pathophysiology of CAD is characterized by chronic formation and progression of atherosclerotic plaques within the coronary arteries, often exacerbated by inflammatory responses [4]. Vulnerable plaques, which are structurally unstable and prone to rupture, are the principal factor driving the progression from sCAD to ACS [5]. Although plaque development typically occurs over decades, inflammation‐driven destabilization can lead to rapid clinical deterioration within a short time frame. Current clinical assessments, including exercise stress testing and imaging techniques such as computed tomography angiography (CTA) or coronary angiography, predominantly evaluate myocardial ischemia or obstructive plaque burden [6]. However, these modalities have limited capability to detect rupture‐prone plaques in asymptomatic individuals. As a result, subclinical coronary atherosclerosis and high‐risk plaques often remain undiagnosed, posing a substantial challenge for ACS prevention. Therefore, early identification of high‐risk individuals and timely therapeutic intervention are essential to prevent plaque rupture, thrombotic vessel occlusion, and the onset of ACS.

The human gastrointestinal tract harbors trillions of microbial communities, collectively known as the gut microbiota. While increasing evidence highlights the critical roles of the gut microbiota in diverse physiological processes, including host metabolism [7] and immune regulation [8], its composition, shaped by both environmental factors and host genetics, has also been implicated in the pathogenesis of CAD [9, 10]. In addition, metabolites produced by gut microbes can enter the host circulation and contribute to the pathology of CAD. Among these, trimethylamine N‐oxide (TMAO) has received considerable attention for its pro‐atherogenic effects [11]. Several investigations have shown that gut microbial profiles [12], either alone or integrated with metabolomic data, can distinguish CAD patients from healthy individuals [13], suggesting that combined microbiome–metabolome profiling may be a valuable approach for CAD risk stratification and early intervention. Nevertheless, most existing studies have focused on nonlethal sCAD and are limited by relatively small sample sizes [10], leaving the specific features of ACS less explored. Recent investigations have identified ACS‐specific biomarkers that can distinguish ACS from healthy and metabolically matched controls [14], but their ability to differentiate ACS from sCAD remains untested. While distinguishing healthy individuals from CAD patients is informative for assessing general cardiovascular risk, the ability to discriminate between sCAD and ACS holds greater clinical importance, as it enables earlier identification of patients at imminent risk and timely intervention to prevent plaque rupture, myocardial infarction (MI), and death.

In this study, we conducted fecal metagenomic and plasma metabolomic analyses in participants classified into three groups: normal coronary arteries (NCA), sCAD, and ACS. We also included a separate group of sCAD patients with a documented history of ACS, providing an opportunity to assess whether gut microbiota composition and metabolite profiles exhibit partial reversion toward non‐ACS patterns after clinical stabilization and to validate ACS‐specific markers identified in our analyses. Together, these observations from a cross‐sectional multi‐omics approach provide insights into the potential involvement of the gut microbial composition and circulating metabolites in CAD progression, providing a basis for future studies to elucidate causality and to explore strategies for early detection and targeted prevention of ACS.

## RESULT

### Study design and baseline characteristics of the cohort

**FIGURE 1:**
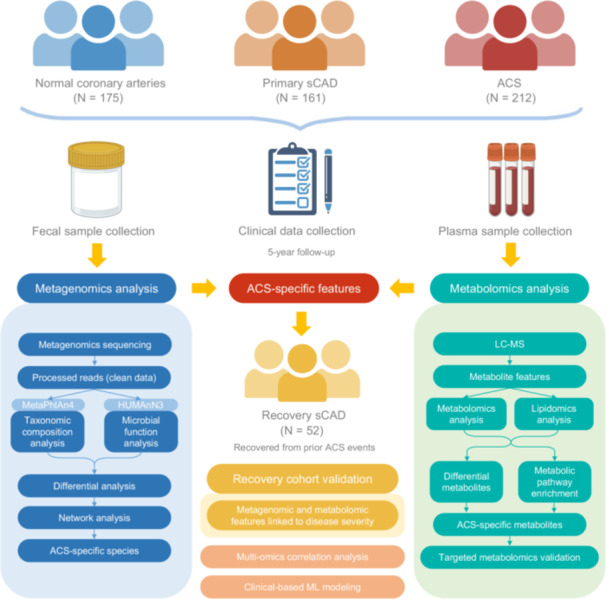
Overview of study workflow and participant cohort. The study cohort comprised four groups: individuals with normal coronary arteries (NCA), patients with primary stable coronary artery disease (sCAD), patients with acute coronary syndrome (ACS), and sCAD patients who transitioned from ACS following revascularization and pharmacological treatments (recovery sCAD). The study followed a comprehensive workflow integrating gut microbiome profiling via metagenomic sequencing and plasma metabolome analysis as a systematic approach to identify robust biomarkers for CAD progression and recovery. The inclusion of recovery sCAD patients provides a valuable framework to investigate the dynamic changes in microbial and metabolic markers, particularly for tracking ACS‐specific features during disease recovery.

Between March 2018 and December 2018, we recruited 548 middle‐aged participants (45–66 years old), including 175 NCA controls, 161 primary sCAD patients, and 212 ACS subjects, at the National Center for Cardiovascular Diseases of China, Fuwai Hospital. Additionally, we included an independent cohort of 52 recovery sCAD patients who had transitioned from ACS to a stable condition following revascularization and pharmacological treatments. To ensure the stability of the primary sCAD cohort, a 5‐year follow‐up was conducted, during which no patients experienced any acute events, confirming their long‐term stability (see Figure 1 for detailed experimental design and workflow). Although body mass index (BMI) levels were comparable across the different groups, primary sCAD patients exhibited clear pathological manifestations of coronary atherosclerosis, making them a more appropriate comparison group for analyzing ACS‐specific gut microbiota and blood metabolic markers. This design allowed for a more accurate evaluation of the critical transition from stable to unstable plaques.

As expected, sCAD and ACS patients had a higher prevalence of comorbid conditions, including hypertension, diabetes, hyperlipidemia, and stable angina pectoris, along with more extensive use of lipid‐lowering therapies compared to NCA controls (all *p* < 0.05) (Table S1). Although blood lipid levels were generally well‐controlled in all three groups, both sCAD and ACS patients exhibited lower total cholesterol (TC), low‐density lipoprotein cholesterol (LDL‐C), and high‐density lipoprotein cholesterol (HDL‐C) levels than NCA controls (all *p* < 0.05), likely reflecting the effects of intensive lipid‐lowering treatment. Fasting blood glucose (FBG) levels were significantly higher in CAD patients compared to NCA controls (both *p* < 0.05), with ACS patients showing the highest levels (7.83 mmol/L), indicating not only a potential stress response to the ACS event but also suboptimal glycemic control. Elevated levels of high‐sensitive C‐reactive protein (hs‐CRP) and N‐terminal pro‐B type natriuretic peptide (NT‐proBNP) in ACS patients (both *p* < 0.001) reflected a pronounced inflammatory state and more severe cardiac dysfunction. Furthermore, lactate dehydrogenase (LDH) levels were significantly higher in ACS patients compared to both NCA and sCAD, suggesting myocardial injury or necrosis (Table S1). Beyond clinical and biochemical parameters, we assessed common lifestyle and dietary variables across all cohorts, including meal patterns, staple–vegetable balance, beverage intake (tea and milk), sleep, and physical activity duration. No significant intergroup differences were observed for these factors (Figure S1, Fisher's exact tests, all *p* > 0.05), suggesting limited lifestyle confounding at the group level.

### Overall gut microbiome profiles in CAD

**FIGURE 2:**
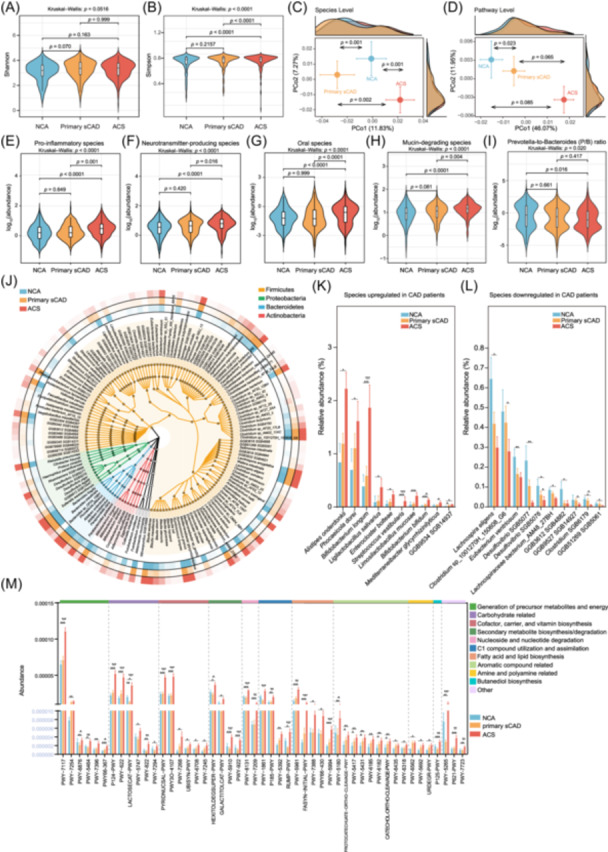
Comprehensive analysis of the changes in gut microbiota in the progression from normal coronary arteries (NCA) to acute coronary syndrome (ACS). Shannon diversity index (A) and Simpson diversity index (B) across the NCA, primary stable coronary artery disease (sCAD), and ACS groups, illustrating the alpha diversity of microbial communities. *p‐*values were computed using the Kruskal–Wallis *H*‐test and the post hoc Dunn's test. (C) Principal coordinate analysis (PCoA) at the species level, showcasing the compositional differences among microbial communities in the NCA, primary sCAD, and ACS groups. (D) PCoA at the pathway level, highlighting the functional differences in microbial metabolic pathways between the groups. PERMANOVA‐based *p*‐values were calculated to assess the differences in group distributions. (E–H) Analysis of the relative abundance of specific microbial species groups, including pro‐inflammatory species, neurotransmitter‐producing species, mucin‐degrading species, and oral species, across the three groups. *p‐*values were computed using the Kruskal–Wallis *H*‐test and the post hoc Dunn's test. (I) Ratio of *Prevotella*‐to‐*Bacteroides* (P/B), a marker of microbial imbalance, showing significant shifts between the NCA, primary sCAD, and ACS groups. *p‐*values were computed using the Kruskal–Wallis *H*‐test and the post hoc Dunn's test. (J) Phylogenetic tree illustrating the evolutionary relationships among the differentially abundant bacteria identified across the groups, providing insights into their taxonomic affiliations. (K) Relative abundance of gut bacterial species that are upregulated in primary sCAD and ACS patients compared to NCA individuals. (L) Relative abundance of gut bacterial species that are downregulated in primary sCAD and ACS patients compared to NCA individuals. (M) Abundance of significantly changed microbial metabolic pathways among the NCA, primary sCAD, and ACS groups as identified by HUMAnN3. In (K–M), significance levels are based on *q*‐values from the MaAsLin2 analysis: *q < 0.1, **q < 0.01, and ***q < 0.001.

Whole‐genome metagenomic sequencing of fecal samples was employed to characterize the microbiota composition and associated functional profiles across different stages of CAD. This technique enabled the detection of 2045 microbial species using the MetaphlAn4 tool and the characterization of 605 functional pathways through HUMAnN3. In contrast to previous reports [15], we observed no statistically significant differences in Shannon diversity among the three groups (Kruskal–Wallis *p* = 0.0516), although there was a trend toward higher values in primary sCAD (*p* = 0.070) and ACS (*p* = 0.163) compared to NCA. In contrast, Simpson diversity differed significantly across groups (Kruskal–Wallis *p* < 0.0001), with ACS showing higher values than both NCA (*p* < 0.0001) and primary sCAD (*p* < 0.0001), while primary sCAD and NCA did not differ significantly (Figure 2A,B). At the species level, microbial composition differed significantly among NCA, primary sCAD, and ACS (all *p* < 0.01, Figure 2C). At the pathway level, a significant difference was observed only between NCA and primary sCAD, while comparisons involving ACS did not reach significance (Figure 2D). We next calculated the total relative abundance of literature‐based, predefined functional groups of taxa classified as being involved in pro‐inflammatory potential, neurotransmitter production, oral origin [16], and mucin‐degradation [17] (Table S2). Compared to NCA and primary sCAD patients, ACS patients showed a more pronounced deviation in their gut microbiome from NCA controls. Compared to both the NCA and primary sCAD groups, ACS patients showed significant increases in pro‐inflammatory species (Figure 2E), neurotransmitter‐producing species (Figure 2F), oral species (many of which are associated with inflammation‐related diseases [18]; Figure 2G), and mucin‐degrading species (Figure 2H) (all comparisons *p* < 0.05). We also examined the abundance of pro‐inflammatory bacteria across the groups and observed a general trend of higher levels in ACS compared to other groups (Figure S2). Finally, we looked at the *Prevotella*‐to‐*Bacteroides* (P/B) ratio, which was previously identified as a reliable predictor of dietary patterns, health conditions, and metabolic functions [19, 20]. Generally, a higher P/B ratio is linked to diets rich in plant‐based fibers and supports beneficial metabolic functions, whereas a lower ratio is often associated with diets higher in animal proteins and fats [20]. Our data reveal that ACS patients, who typically exhibit an inflammatory status, also show a lower P/B ratio compared to NCA group (*p* = 0.016) (Figure 2I).

We then sought to identify individual gut microbial features associated with the spectrum of CAD, in particular features that show consistent alteration with disease severity. To do so, we utilized PERMANOVA and MaAsLin2 to assess and correct for the impact of clinical variables (age, sex, medication usage, smoking history, and BMI) on microbial community composition [14], thereby differentiating biological differences from confounding influences. Using a stringent filtering approach (MaAsLin2 with FDR‐adjusted *q* < 0.1 for all intergroup comparisons), we identified 150 differentially abundant taxa across the NCA, primary sCAD, and ACS groups (see phylogenetic cladogram in Figure 2J). Among these taxa were 28 bacterial species with progressively increasing abundance and 15 species with progressively decreasing abundance (Table S3). The top 10 species in each category, ranked by relative abundance, are shown in Figure 2K,L. Notably, taxa such as *Alistipes onderdonkii*, *Bifidobacterium* spp., and *Streptococcus* spp. are prominently upregulated, and many of these species are known to contribute to pro‐inflammatory processes through pathways such as lipopolysaccharide production or immune activation [21, 22]. Conversely, species such as *Lachnospiraceae* spp., *Clostridium* spp., and *Eubacterium ventriosum* that have been associated with anti‐inflammatory or gut‐barrier‐supporting roles [23, 24] were significantly downregulated. This microbial stratification not only highlights the dynamic differences in gut microbiota between groups, but it also underscores the dominance of pro‐inflammatory bacterial signatures as a hallmark of ACS patients.

We further inspected the functional aspects of the ACS‐ and sCAD‐associated gut microbiome. Of the 605 pathways identified by HUMAnN3, we selected 49 significantly altered pathways (*q* < 0.1, fold change >1.5 or <0.6, average abundance >3e‐5) for visualization (Tables S4, 5 and Figure 2M). Generally, ACS patients were uniquely enriched with gut microbial pathways involving fatty acids and ketogenesis, formaldehyde and phenol metabolism, and mevalonate‐related metabolism. These findings not only illustrate significant functional alterations in the gut microbiome associated with CAD progression but also indicate increased levels of lipids and ketone bodies, as well as a potentially enhanced oxidative metabolism in ACS patients. Together, these findings provide critical insights into the gut−CAD axis, emphasizing the potential of microbial biomarkers in cardiovascular pathology.

### Plasma metabolomic and lipidomic profiles in CAD

**FIGURE 3:**
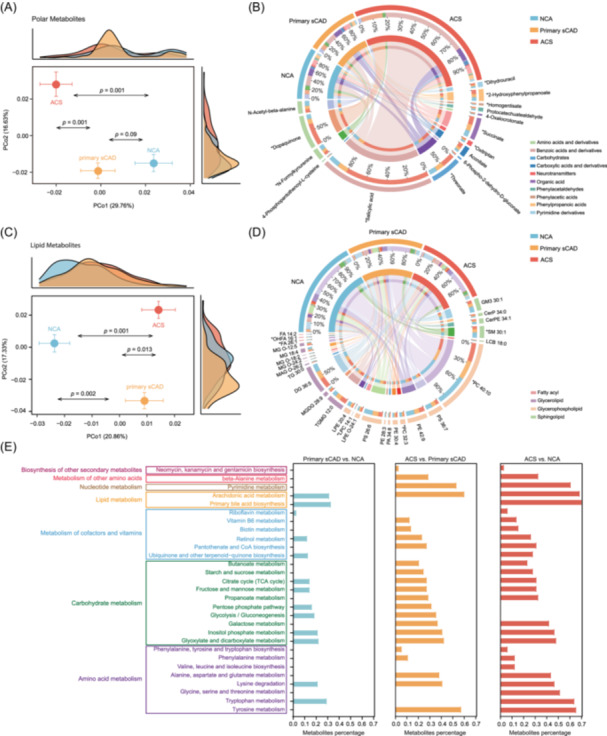
Metabolomic and lipidomic profiles associated with coronary artery disease (CAD) progression. (A) Principal coordinate analysis (PCoA) of polar metabolites depicting the variation in the overall metabolic profile across the groups. PERMANOVA‐based *p*‐values were calculated to assess the differences in group distributions. (B) Circos plot illustrating the composition and distribution of polar metabolites in the three groups. The outermost ring is divided into two sections. The upper half represents the three study groups (NCA, primary sCAD, and ACS). The lower half represents different metabolite classes. Inner ribbons indicate the relative proportion of each metabolite class in each group, or conversely, the proportion of each group within a given metabolite class. Connecting lines within the plot highlight associations between specific metabolite classes and their distribution across different groups. The legend (bottom‐right) denotes the different metabolite categories. (C) PCoA of lipid metabolites depicting the variation in the overall lipid profile across the groups. PERMANOVA‐based *p*‐values were calculated to assess the differences in group distributions. (D) Circos plot showing the distribution of lipid metabolites across the three groups. Similar to (B), the outermost ring consists of two sections. The upper half represents the three study groups, while the lower half represents different lipid subclasses. Inner ribbons illustrate the relative proportion of each lipid subclass in each group, or the proportion of each group within a given lipid subclass. Connecting lines highlight the relationships between specific lipid subclasses and their distribution across different groups. The legend (bottom‐right) represents the different lipid categories. (E) KEGG pathway enrichment analysis of significantly altered metabolites among the three groups. Bar plots show the percentage of metabolites enriched in each KEGG pathway, separately for the three pairwise comparisons: primary sCAD versus normal coronary arteries (NCA), acute coronary syndrome (ACS) versus primary sCAD, and ACS versus NCA. Pathways are grouped by functional categories and listed along the *y*‐axis. Enrichment was performed using MetaboAnalyst based on compound classification and KEGG annotation.

To further examine the metabolic shifts in CAD, we conducted plasma untargeted metabolomics and lipidomics among the NCA, primary sCAD, and ACS groups to identify factors contributing more directly to the development of CAD. Untargeted polar and lipidomic metabolome analysis using ultra‐performance liquid chromatography coupled with high‐resolution mass spectrometry (UPLC‐HRMS) was first performed on a subset of 271 individuals (91 NCA, 78 primary sCAD, and 102 ACS) (see Methods and Tables S6, 7 for details). The NCA, primary sCAD, and ACS groups displayed a clear separation based on the profile of polar metabolomes (Figure 3A). Based on a significance threshold of *q* < 0.1 (MaAsLin2 analysis for all intergroup comparisons), we identified 21 significantly changed metabolites in NCA versus primary sCAD, 51 in NCA versus ACS, and 38 in primary sCAD versus ACS (Table S6). Of these, 15 metabolites showed a fold change >1.2 or <0.8 (Figure 3B, Figure S3). Specifically, 11 metabolites belonging to organic acid, carbohydrates, carboxylic acids, phenylpropanoic acids, phenylacetic acids, benzoic acids, and pyrimidine derivatives were notably increased in CAD patients compared to NCA individuals, and this trend became more pronounced with increasing disease severity. Conversely, four metabolites belonging to amino acids, phenylacetaldehydes, and neurotransmitters were significantly decreased in CAD patients (Figure 3B). Notably, there was a group of metabolites that were dramatically elevated in ACS patients, including 2‐hydroxyphenylpropanoate, dihydrouracil, succinate, threonate, salicylic acid, homogentisate, and dopaquinone (Table S6 and Figure 3B). Hydroxybutyrate is a typical ketone, and hydroxyphenylpropanoate belongs to the homologues of phenol, and this increase is in line with our functional pathway analysis results. Additionally, increased plasma levels of phenylalanine and succinate are known to be associated with chronic inflammation status [25, 26, 27, 28].

Similarly, the plasma lipidomes of the NCA, primary sCAD, and ACS groups were significantly different (Figure 3C). A total of 149 lipid metabolites were identified and classified into four categories: 27 fatty acids (FAs), 38 glycerolipids (GLs), 69 glycerophospholipids (GPs), and 15 sphingolipids (SLs) (Table S7). At *q* < 0.1 (MaAsLin2 analysis for all intergroup comparisons), 103 differential metabolites were identified when comparing NCA versus primary sCAD, 91 when comparing NCA versus ACS, and 107 when comparing primary sCAD versus ACS (Table S7), with 28 lipids showing fold changes >1.2 or <0.8 (Figure 3D, Figure S3). In CAD patients, 11 lipids (5 GLs, 4 GPs, and 2 SLs) were significantly increased, while 17 lipids (4 GLs, 7 GPs, 3 SLs, and 3 FAs) were markedly decreased (Figure 3D). Specifically, triacylglycerols (TG 30:0 and TGMG 12:0), diacylglycerols (DG 36:5), lyso‐glycerophospholipids (LPC 14:1 and LPE O‐24:1), monoacylglycerols (MG O‐12:5 and MGDG 28:9), sphingolipids (LCB 18:0), glycerophosphates (PA 34:8), ceramides (CerP 34:0), and glycerophosphoethanolamines (PE 30:4) were notably increased in primary sCAD and ACS patients compared to NCA individuals (Figure 3D and Table S7). A previous study reported that ceramide accumulation in obese or dyslipidemic individuals can lead to tissue dysfunction, which is associated with diabetes and cardiovascular disease [29]. Moreover, sphingolipids can be selectively increased by inflammatory agonists by activating enzymes in the de novo ceramide biosynthesis pathway. TGs also play a crucial role in the activation of inflammatory macrophages; in the production of inflammatory mediators such as IL‐1β, IL‐6, and PGE2; and in phagocytic capacity [30]. Therefore, CAD patients, particularly ACS patients, present a typical inflammatory lipid metabolite profile.

Metabolite set enrichment analysis based on MetaboAnalyst was performed among the differential metabolites (MaAsLin2 *q* < 0.1), and compared between every two groups. A bar plot reveals that the amino acid metabolism pathway (phenylalanine, tyrosine and tryptophan biosynthesis; phenylalanine metabolism; tyrosine metabolism) and carbohydrate metabolism (galactose metabolism, propanoate metabolism, starch and sucrose metabolism, butanoate metabolism) were enriched in ACS patients compared to the NCA and primary sCAD groups (Figure 3E), which was consistent with their critical role in immunoregulation [28].

### ACS‐specific microbial and metabolite characteristics

**FIGURE 4:**
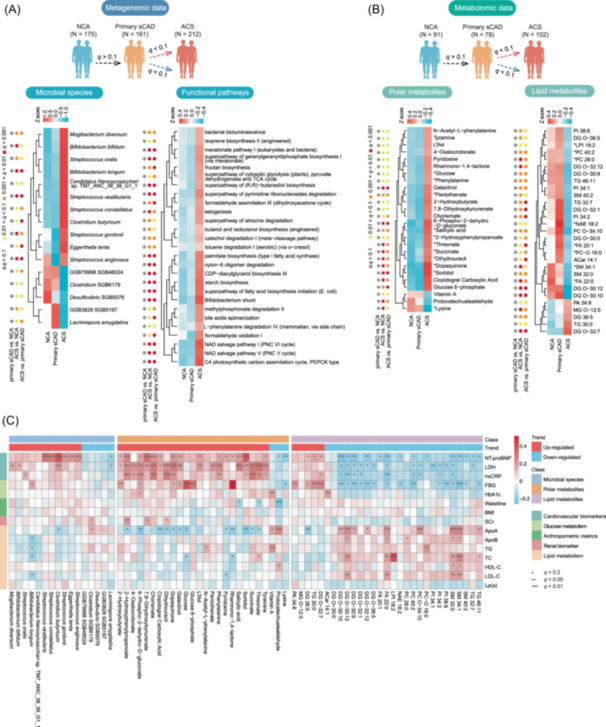
Acute coronary syndrome (ACS)‐specific alterations in gut microbiota and metabolites. (A) Heatmap showing the relative abundance of ACS‐specific bacterial species and microbial metabolic pathways across the different groups. (B) Heatmap showing the relative abundance of ACS‐specific polar and lipid metabolites across the different groups. In the schematic diagrams above A and B, red arrows indicate significant upregulation, blue arrows indicate significant downregulation, and black arrows indicate no significant change. Heatmap color intensity in the heatmap indicates the z‐score of relative abundance (red = high, blue = low). ACS‐specific bacterial species or metabolites are defined as features without significant differences between NCA and primary stable coronary artery disease (sCAD) but with significant differences between primary sCAD and ACS. The dot plot to the left of each heatmap displays the *q*‐values from pairwise differential abundance analysis (MaAsLin2), highlighting statistical significance. * indicates metabolites identified by UPLC‐MS/MS. (C) Heatmap of the Spearman's rank correlation coefficient of 16 significant taxa, 26 polar metabolites, and 29 lipid metabolites and clinical indices. Red squares indicate positive associations; blue squares indicate negative associations. *p*‐values were adjusted using the Benjamini–Hochberg (BH) method. Statistical significance is indicated within the squares (*q < 0.2, **q < 0.05, ***q < 0.01). NCA, normal coronary arteries.

ACS poses a much greater threat to patients than sCAD, making it critical to identify ACS‐specific features that contribute to its progression. In our analysis, we identified gut microbial taxa, functional pathways, and metabolites that were specifically altered in the ACS group (MaAsLin2 *q*_primary sCAD vs. NCA_ > 0.1, *q*_ACS vs. NCA/primary sCAD_ < 0.1). These features, which are significantly enriched or depleted in ACS, may play key roles in plaque destabilization and rupture (Figure 4A,B).

We found 11 gut bacteria significantly elevated in ACS patients, including *Streptococcus* spp. (*S. anginosus*, *S. oralis*, *S. constellatus*, *S. gordonii*, and *S. vestibularis*), *Bifidobacterium* spp. (*B. bifidum* and *B. longum*), *Mogibacterium diversum*, and *Eggerthella lenta*. Conversely, species such as *Lacrimispora amygdalina*, *Clostridium* spp., and *Desulfovibrio* spp. were significantly reduced (Table S8 and Figure 4A). We systematically evaluated the effect of sex on the significant bacteria taxa using generalized linear models. Only one microbe, GGB79998 SGB48024, showed a significant sex interaction (*p* = 0.024), with a negative association in males (*β* = −4.92) reversing to a positive association in females (*β* = 11.65). Additionally, ACS patients showed enrichment of functional pathways involved in ketogenesis, fatty acid metabolism, and bile acid biosynthesis (Table S9 and Figure 4A).

We also investigated ACS‐specific alterations in polar metabolites and lipidomics (Figure 4B and Table S10). Among the polar metabolites, 2‐hydroxybutyrate (2‐HB) showed a significant increase. Recent research suggests that 2‐HB might act as a cardioprotective factor and biomarker of tissue ischemia, indicating its potential relevance in the context of ACS [31]. Phenylalanine and its derivative N‐acetyl‐L‐phenylalanine were both significantly elevated in the ACS group. These metabolites can act through adrenergic receptors, potentially influencing cardiovascular outcomes [32]. Tyramine, succinate, pantothenate, glucose, rhamnono‐1,4‐lactone, and other metabolites were also significantly elevated. Conversely, reductions of lysine, an essential amino acid crucial for protein synthesis [33]; protocatechuate aldehyde, a phenolic compound with antioxidant properties [34]; and vitamin A, known for its role in immune function and anti‐inflammatory properties [35], in the ACS group suggest metabolic imbalances and heightened catabolic states, potentially indicating compromised antioxidant defenses (Figure 4B, Figure S3). Additionally, we observed distinct lipidomic profiles in the ACS group: DG O‐32:7, TG 30:0, DG O‐36:5, MG O‐12:5, and PA 34:8 were significantly elevated, whereas other lipids, including various phosphatidylcholines (PCs) and phosphatidylinositols (PIs), were generally reduced (Figure 4B and Table S10). This pattern suggests alterations in lipid metabolism that may contribute to ACS pathophysiology, reflecting changes in energy storage, membrane composition, and signaling pathways [13]. The elevation of specific diacylglycerols and triacylglycerols may indicate increased lipolysis and re‐esterification processes, while the reduction in other lipids could be linked to impaired lipid transport and metabolism [36].

We further analyzed the associations between ACS‐specific features (microbial taxa, lipid metabolites, and polar metabolites) and clinical indicators (Figure 4C). As expected, the ACS‐specific features exhibited strong correlations with cardiovascular biomarkers, further emphasizing their relevance to CAD progression. Notably, several gut microbial alterations, including changes in *Streptococcus* spp., *Clostridium butyricum*, and *Mogibacterium diversum*, showed significant associations with specific cardiovascular biomarkers such as NT‐proBNP, hs‐CRP, and LDH. These findings suggest a potential link between gut microbiota and the systemic inflammation, cardiac dysfunction, and tissue injury observed in CAD progression. Lipid metabolites also demonstrated strong correlations with lipid‐metabolism‐related clinical indicators such as ApoB, LDL‐C, and triglycerides, reinforcing the importance of lipid metabolism in ACS pathophysiology. Polar metabolites displayed notable associations with markers of tissue injury and metabolic stress. LDH, in particular, showed strong correlations with 2‐HB, succinate, and other intermediates of energy metabolism, suggesting a metabolic shift during ACS events.

### Restoration of gut microbiota and metabolites following ACS recovery

**FIGURE 5:**
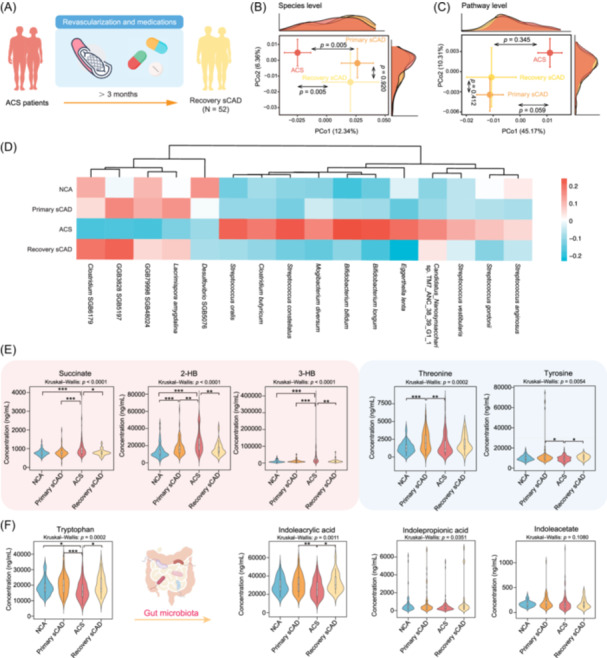
Recovery of microbiota and metabolites in stable coronary artery disease (sCAD) and their relevance to acute coronary syndrome (ACS). (A) Schematic representation of the characteristics of ACS patients and recovery sCAD patients. PCoA of (B) microbial community and (C) microbial metabolic pathways in recovery sCAD patients reveals a microbial profile closer to primary sCAD and distant from ACS. PERMANOVA‐based *p*‐values were determined to assess the differences in group distributions. (D) Recovery of ACS‐specific bacteria in recovery sCAD patients, where all ACS‐specific bacteria are restored to levels similar to those in primary sCAD. (E) Circulating metabolites elevated in ACS (e.g., succinate, 2‐HB, 3‐HB) decreased significantly in the recovery group, whereas metabolites reduced in ACS (e.g., threonine, tyrosine) increased significantly. (F) Tryptophan‐ and indole‐metabolism‐related metabolites suppressed in ACS were also restored in recovery sCAD. *p‐*values were computed using the Kruskal–Wallis *H*‐test and the post hoc Dunn's test. Asterisks indicate significance level: *p < 0.05, **p < 0.01 and ***p < 0.001. PCoA, principal coordinate analysis.

To determine whether ACS‐associated bacteria and metabolites represent intrinsic features that change with CAD severity, we recruited an independent cohort of recovery sCAD patients (*N* = 52) with a history of ACS who had transitioned to sCAD following interventional and pharmacological treatments for a minimum of 3 months. We compared gut microbiota and blood metabolites between recovery sCAD, primary sCAD, and ACS patients to identify recovery‐associated profiles (Figure 5A).

Baseline analysis showed that recovery sCAD and primary sCAD patients exhibited similar physiological phenotypes, although a higher proportion of recovery sCAD patients were treated with statins and antiplatelet drugs. This was reflected in improved serum lipid profiles in the recovery sCAD group compared to the primary sCAD patients (Table S11). A comparison of gut microbiota composition (Figure 5B) and metagenomic pathway profiles (Figure 5C) across the primary sCAD, ACS, and recovery sCAD groups revealed that recovery sCAD patients shared significant similarities with primary sCAD patients, with both groups showing a clear departure from ACS. This trend was further reflected in the abundance patterns of ACS‐specific bacterial species across the three groups (Figure 5D). As patients transitioned from ACS to recovery sCAD, we observed a clear shift in microbial composition, indicating a partial reversal of the ACS‐associated gut dysbiosis. Notably, pro‐inflammatory *Streptococcus* spp., which were enriched in ACS patients, showed a marked decreasing trend in recovery sCAD (Figure 5D).

To further validate the changes in abundance of ACS‐specific metabolites identified in our previous screening, we conducted targeted metabolomics analysis across the four groups (NCA, primary sCAD, ACS, and recovery sCAD). This allowed us to confirm the metabolic shifts and provided additional insights into the interplay between metabolites and microbiota during CAD progression and recovery. Our analysis revealed two distinct patterns of metabolite changes: Some metabolites elevated in ACS decreased during recovery, while others reduced in ACS increased upon recovery (Table S12). We first examined previously reported gut‐microbiota‐derived metabolites associated with CAD, such as TMAO, phenylacetylglutamine (PAGln), and their upstream and downstream products. Consistent with prior studies, TMAO and PAGln were significantly elevated in CAD patients compared to NCA controls [11, 32]. However, in our data, these metabolites showed no significant differences between the ACS and sCAD groups, failing to distinguish stable and acute CAD states (Figure S4). This observation highlights the need to identify novel metabolites that can be used to monitor disease progression and recovery. In contrast, the ACS‐specific metabolites from our analysis exhibited distinct trends that could track disease dynamics. Three metabolites, succinate, 2‐HB, and 3‐HB, were significantly elevated in ACS patients compared to both sCAD groups (Figure 5E, red box), and their levels decreased upon recovery. Conversely, threonine and tyrosine, which were markedly reduced in ACS patients, increased in the recovery group (Figure 5E, blue box). Microbiome‐derived tryptophan‐related metabolites, including indoleacrylic acid and indolepropionic acid, also demonstrated similar recovery trends, with concentrations in recovery sCAD patients reversing the reductions observed in ACS patients (Figure 5F).

Overall, our findings demonstrate that specific gut microbial and metabolic signatures identified in ACS can partly reverse during recovery, supporting their potential utility as indicators for both disease progression and resolution.

### Multi‐omics correlation analysis reveals the microbiota‐metabolite‐pathway links in ACS pathophysiology

**FIGURE 6:**
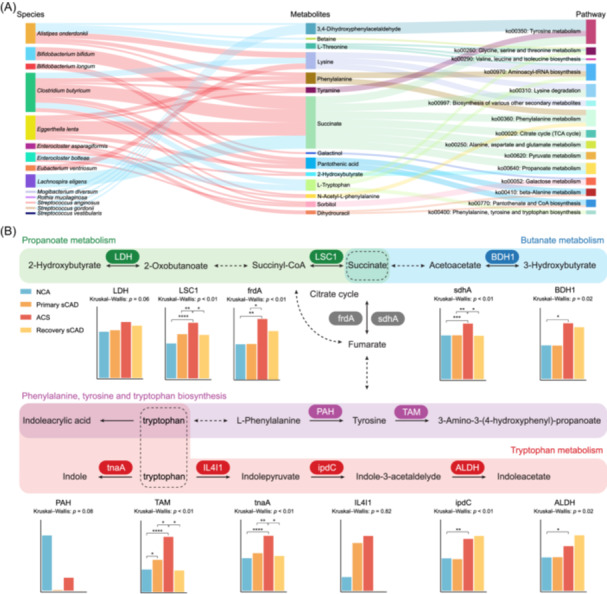
Multi‐omics correlation analysis revealing microbiota−metabolite−pathway interplay. (A) Sankey diagram of microbiota−metabolite−pathway based on MetOrigin analysis results. Diagram illustrates the complex interplay between the microbial community and the metabolic profile. The first column (left) represents the acute coronary syndrome (ACS)‐specific bacteria or the bacteria with continuous increase/decrease identified in Figure 2 that can metabolize (produce or consume) key metabolites. The second column (middle) denotes the ACS‐specific and target‐validated metabolites. The third column (right) indicates the metabolic pathways in which these metabolites are involved. Connections between the first and second columns are color‐coded: red lines signify that the bacteria can produce the corresponding metabolite, while blue lines indicate that the bacteria can consume the metabolite. (B) Network diagram referencing KEGG. The network diagram shows the abundance of key enzymes identified from metagenomic data in the metabolic processes of ACS‐specific key metabolites. *p‐*values were computed using the Kruskal–Wallis *H*‐test and the post hoc Dunn's test. Asterisks indicate significance levels: *p < 0.05, **p < 0.01, ***p < 0.001, and ****p < 0.0001. LDH: L‐lactate dehydrogenase [EC:1.1.1.27]; LSC1: succinyl‐CoA synthetase (ADP‐forming) [EC:6.2.1.5]; frdA: fumarate reductase [EC:1.3.5.4; merged into EC:1.3.5.1 in updated Enzyme Commission number system]; sdhA: succinate dehydrogenase [EC:1.3.5.1]; BDH1: 3‐hydroxybutyrate dehydrogenase [EC:1.1.1.30]; PAH: phenylalanine‐4‐hydroxylase [EC:1.14.16.1]; TAM: MIO‐dependent L‐tyrosine 2,3‐aminomutase [EC:5.4.3.6]; tnaA: tryptophanase [EC:4.1.99.1]; IL4I1: L‐amino‐acid oxidase [EC:1.4.3.2]; ipdC: indolepyruvate decarboxylase [EC:4.1.1.74]; ALDH: aldehyde dehydrogenase (NAD+) [EC:1.2.1.3].

To elucidate the microbial and metabolic interplay driving ACS pathogenesis, we integrated metagenomic and metabolomic data through a multi‐omics framework. Using MetOrigin [37], a dual‐dimensional analysis tool that combines biological relevance and statistical correlation, we traced the origins of key metabolites to specific gut microbes. Our analysis identified several metabolites of interest (Figure 6A), particularly 2‐HB and succinate, which were significantly elevated in ACS patients and subsequently declined in recovery sCAD. Both metabolites are involved in propionate metabolism. Notably, their levels correlated strongly with specific bacterial species, including *Clostridium butyricum*, *Enterocloster bolteae*, *Bifidobacterium bifidum*, *Lachnospira eligens*, and *Eggerthella lenta*. In contrast, metabolites that were significantly reduced in ACS but restored in recovery sCAD, such as tryptophan, which is involved in the phenylalanine, tyrosine, and tryptophan biosynthesis pathway, exhibited a strong association with *Enterocloster bolteae* and *Clostridium butyricum*. Further correlation analysis revealed that most bacterial species exhibited a positive correlation with succinate and 2‐HB (red connections in the in Figure 6A) but a negative correlation with tryptophan (blue connections in Figure 6A).

Functional profiling with HUMAnN3 revealed a significant enrichment of enzyme‐coding genes related to ketogenesis, central carbon metabolism, and amino acid metabolism in ACS patients. Notably, genes encoding key enzymes for 3‐HB and succinate biosynthesis, including BDH1 (3‐hydroxybutyrate dehydrogenase, EC:1.1.1.30), frdA (fumarate reductase, EC:1.3.5.4), and sdhA (succinate dehydrogenase, EC:1.3.5.1), exhibited higher relative abundances in ACS compared to the NCA and/or sCAD groups (*p* < 0.05; Figure 6B), in line with the elevated plasma levels of 3‐HB and succinate we observed in ACS patients (Figure 5E). Additionally, the tryptophan‐catabolizing enzyme tnaA (tryptophanase, EC:4.1.99.1) was significantly enriched in ACS (*p* < 0.01), consistent with their reduced plasma tryptophan levels, consistent with the observed decrease in tryptophan levels within this group.

Mediation analysis further explored how gut microbes might influence metabolites through functional microbial pathways, and how these metabolites subsequently associated with ACS (Figure S5 and Table S13). Figure S5A demonstrates short‐chain fatty acid metabolism, amino acid metabolism, and central carbon metabolism have acted as mediators linking microbial shifts to changes in levels of ACS‐specific metabolites. In Figure S5B, these metabolites partly mediated the relationships between specific taxa and ACS status, consistent with our earlier observations that ACS was characterized by elevated *Clostridium butyricum*, higher 2‐HB and 3‐HB, and reduced *Clostridium* SGB6179 and threonine.

Together, these findings, integrating multi‐omics evidence, support the notion that gut microbiota may influence CAD through metabolite‐mediated pathways.

### Integrating clinical, microbial, and metabolic features to distinguish different CAD stages using machine learning models

Having observed trends of microbial and metabolic changes alongside disease development and recovery, we hypothesized that those signals might be valuable as disease biomarkers. To better align with clinical practice, we constructed machine learning models incorporating established cardiovascular risk factors [14] supplemented with gut microbiome signatures, targeted quantified metabolites, and an integrated combination of these three dimensions. By integrating these multi‐dimensional features, we aimed to assess whether microbial and metabolic profiles could provide additional value in distinguishing different stages of CAD. The inclusion of these features offered the potential to uncover subtle, disease‐specific patterns that might not be detected by traditional markers alone. For this analysis, we split the data set into a discovery data set and a hold‐out data set in a stratified 8:2 ratio. The discovery set was further classified into training and validation sets for 10‐fold cross‐validation. The hold‐out data set was utilized as test data to assess model robustness and generalizability.

**FIGURE 7:**
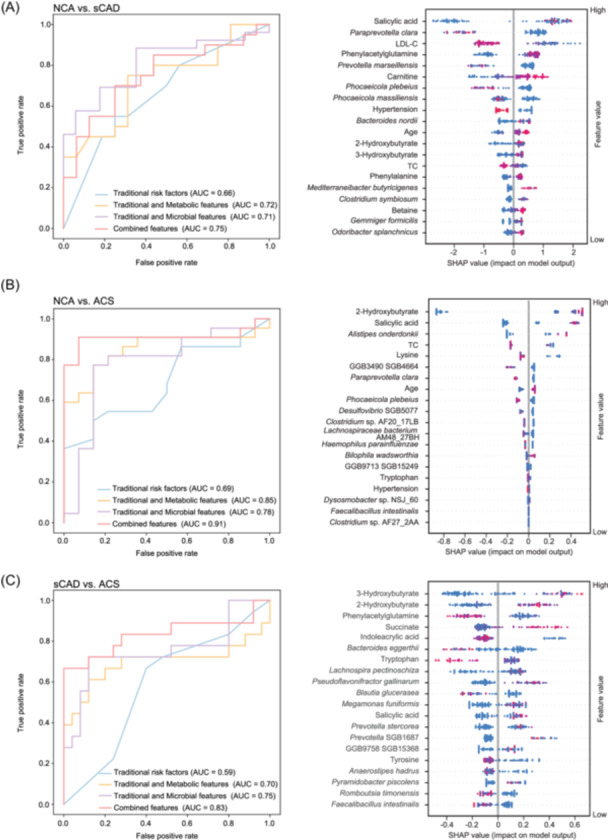
Classification models for different stages of coronary artery disease (CAD) based on clinical indicators, microbial characteristics, and metabolic features built using the Light Gradient Boosting Machine (LightGBM) machine learning algorithm. (A) Classification model differentiating normal coronary arteries (NCA) fromstable CAD (sCAD). The Boruta algorithm was used to select features. The ROC curves show the AUC of the model when applied to the independent hold‐out dataset, while the SHAP plots illustrate the contribution of each feature to the combined model. The Top 20 features are ranked according to their importance, as determined by the |SHAP| value, with color and intensity indicating the direction and magnitude of their contribution to the model's output. (B) Classification model differentiating NCA and ACS. (C) Classification model differentiating sCAD and ACS.

As shown in Figure 7, evaluation of classification performance indicated that employing only traditional clinical markers as features resulted in limited diagnostic performance. By integrating metabolic and microbial features (Figure S6), we constructed models that demonstrated marked improvements in accuracy. The combined models, validated on the hold‐out data set, achieved AUCs of 0.75 for distinguishing NCA from sCAD (Figure 7A), 0.91 for distinguishing NCA from ACS (Figure 7B), and 0.83 for distinguishing sCAD from ACS (Figure 7C). Notably, the combined model excelled in distinguishing sCAD from ACS, whereas traditional markers showed limited discriminative power (AUC = 0.59). The key features driving this improved performance were all metabolic and microbial, including 2‐HB, 3‐HB, PAGln, succinate, *Bacteroides eggerthii*, *Lachnospira pectinoschiza*. These findings highlight the unique ability of microbial and metabolic profiles to capture dynamic changes between stable and acute CAD states, underscoring their potential as biomarkers for monitoring disease progression and enabling precise stratification.

## DISCUSSION

As CAD leads to millions of deaths annually, a thorough understanding of its etiology and the establishment of early warning approaches are crucial for combating this disease. In this study, we examined the altered profiles of the gut microbial community and blood metabolites in ACS patients compared to both NCA controls and sCAD patients. We highlighted the relationship between pro‐inflammatory bacteria, metabolites, and CAD severity, identifying key microbial and metabolic features that change with CAD progression and tend to revert toward stable levels following recovery from ACS. Furthermore, classifiers based on the metagenomic and metabolomic profiles exhibited promising accuracy in distinguishing patients with different CAD phenotypes, particularly for identifying ACS patients.

Inflammation is a hallmark feature of CAD, with hs‐CRP recognized as a key predictor of cardiac events, independent of lipid levels [38, 39, 40]. In both ACS and sCAD patients, the gut microbiota similarly exhibits a pro‐inflammatory profile characterized by an increase in pro‐inflammatory and oral‐origin bacterial species. For example, several inflammation‐prone *Streptococcus* spp. were significantly elevated in ACS patients. These species are linked to enhanced inflammatory responses and may contribute to the destabilization of atherosclerotic plaques [22, 41], exacerbating the inflammatory milieu in ACS patients. Our analysis revealed that *Alistipes putredinis* was significantly increased in ACS compared to NCA, suggesting it plays a pivotal role in promoting a pro‐inflammatory state. *Alistipes* spp., including *A. putredinis*, can induce inflammation through lipopolysaccharide production, which triggers immune responses and systemic inflammation [21]. We also observed that some species commonly recognized to be beneficial, such as *Bifidobacterium*, were also elevated in sCAD and ACS patients compared to NCA controls. These bacteria are known producers of succinate [42], a gut microbial metabolite linked to a pro‐inflammatory and oxidative stress pattern and higher CAD risk [43]. Additionally, *Bifidobacterium* possess mucin‐degrading enzymes [17, 24]. In vulnerable patients, a significant increase in these bacteria may compromise the intestinal mucosal barrier, raising the risk of gut permeability (‘leaky gut’), which in turn can trigger and exacerbate inflammation [17, 24]. However, *Bifidobacterium* exhibited substantial functional diversity, with effects being both strain‐specific and context‐dependent. While certain strains may contribute to succinate accumulation or mucin degradation, others are well known for their anti‐inflammatory, immunomodulatory, and gut barrier‐supporting functions [44, 45]. The elevation observed in ACS may therefore reflect strain‐level compositional shifts or functional adaptation shaped by the disease‐modified intestinal microenvironment.

In accordance with the pro‐inflammatory gut microbiota profile, CAD patients showed enrichment in serum metabolites that promote inflammation. As noted above, succinate, a key intermediate in the tricarboxylic acid (TCA) cycle, has been recognized for its multifaceted role in cardiovascular diseases [46]. Recent research highlights that there is an intricate interplay between gut microbiota and cardiovascular health, with succinate emerging as a critical gut‐derived metabolite that can influence host metabolism [46]. Additionally, we observed the elevation of phenylalanine and its catabolites (phenyllactic acid and PAGln) in CAD patients. Usually, phenylalanine is metabolized into phenylpyruvate via microbial metabolic pathways, which further produces phenyllactic acid. This compound combines with glutamine in the liver to produce PAGln [32]. Recent reports have identified phenylalanine and its metabolites as key oxidative and pro‐inflammatory factors contributing to the progression of cardiac disease [32, 47, 48]. We also observed a significant elevation of TMAO and its precursors, carnitine and betaine, in CAD patients, consistent with previous studies that validated their role as markers of atherosclerotic burden [11, 49]. However, the lack of significant differences between the sCAD and ACS groups suggests that while these metabolites are indicative of atherosclerosis, they may not be reliable markers for distinguishing between clinically stable and acute CAD states.

To identify ACS markers that are more specific, we focused on metabolites that showed significant alterations in the ACS group. Among these, succinate, 2‐HB, and 3‐HB were particularly noteworthy. 2‐HB and 3‐HB are common intermediate metabolites of protein and lipids. While 3‐HB serves as a major ketone body and alternative energy source during fatty acid β‐oxidation, exogenously supplemented or endogenously 3‐HB supplementation has demonstrated protective cardiometabolic effects, including reducing FBG, improving insulin sensitivity, and mitigating atherosclerosis [50, 51]. Therefore, its elevation may be a compensatory consequence of stress‐induced hyperglycemia or glucocorticoid‐driven ketogenesis [52, 53, 54], rather than a direct pathogenic effect. In contrast, 2‐HB originates primarily from the reduction of α‐ketobutyrate (αKB) in the cytoplasm. Under physiological conditions, mitochondrial αKB undergo oxidative decarboxylation via the branched‐chain α‐keto acid dehydrogenase complex to generate succinyl‐CoA for TCA cycle [55]. However, pathological states, such as oxidative stress and increased fatty acid oxidation induced by insulin resistance, elevate the mitochondrial NADH/NAD+ ratio, potently inhibiting the activity of the aforementioned dehydrogenase complexes. This impairment diverts αKB toward cytoplasmic reduction to 2‐HB [55]. Elevated 2‐HB closely associates with insulin resistance severity, deterioration of impaired glucose tolerance, and the risk of progression to type 2 diabetes [56] and CAD [57]. Although the effects of 2‐HB and 3‐HB on ACS pathogenesis remain to be fully elucidated, their distinct biological roles, despite near‐identical structures warrant in‐depth investigation. In addition to these elevated metabolites, several amino acids, including threonine, tyrosine, and tryptophan, were significantly reduced in ACS. Notably, indoleacrylic acid, a gut microbiota‐derived metabolite of tryptophan, also showed a significant decrease. Together, these ACS‐specific metabolic shifts, with increases in oxidative stress‐related metabolites and decreases in selected amino acids and microbiota‐derived compounds, provide potential biomarkers for distinguishing ACS from stable CAD and offer insights into disease mechanisms.

Having identified microbiome and metabolite signatures characteristic of ACS, we sought to determine whether these features would also revert toward stable CAD levels during recovery. To investigate this, we compared patients who had transitioned from ACS to a stable condition with those who had primary sCAD, enabling us to evaluate recovery‐associated shifts in microbial composition and circulating metabolites. Intriguingly, the ACS‐specific gut microbiota and metabolites in these patients also reversed, including pro‐inflammatory bacteria *Streptococcus* spp. (*S. anginosus*, *S. constellatus*, *S gordonii*, *S. oralis*, and *S. vestibularis*) and succinate‐producing bacteria *Bifidobacterium* spp. (*B. bifidum* and *B. longum*). Meanwhile, the increase of 2‐HB, 3‐HB, and succinate in ACS patients was dramatically reversed during disease recovery. These findings highlight that gut microbiota and associated metabolites differ markedly across clinical stages of CAD, suggesting their potential to reflect shifts in disease state [58].

In addition to microbial and metabolic profiling, our study revealed a significant enrichment of microbial enzyme functions associated with key metabolic pathways in ACS patients. Notably, enzyme families such as frdA (fumarate reductase, EC:1.3.5.4), sdhA (succinate dehydrogenase, EC:1.3.5.1), and BDH1 (3‐hydroxybutyrate dehydrogenase, EC:1.1.1.30) showed higher relative abundances in ACS. These enzymes participate in pathways producing pro‐inflammatory metabolites like succinate and hydroxybutyrates, which were significantly elevated in ACS patients compared to both the sCAD and NCA groups. The enrichment of these enzymatic functions aligns with the observed metabolic shifts, providing mechanistic insights into how microbial metabolism may contribute to metabolic disturbances in CAD.

Given the limitations in the current early warning systems for ACS, there is an urgent need to improve risk prediction and prognosis at the individual, family, community, and pre‐hospital levels. Better identification of individuals at risk for ACS can significantly alleviate the public health burden associated with CAD. In this study, we utilized the LightGBM algorithm to combine metagenomic and metabolomic data to identify potential biomarkers for ACS risk stratification. While clinical metadata alone showed limited discriminative power for disease stratification, its integration with microbiome and metabolomic features significantly enhanced model performance. Notably, the combined models demonstrated exceptional performance in distinguishing ACS patients from both NCA controls and sCAD patients, offering a robust framework for precise risk stratification and potential therapeutic targeting. Moreover, earlier metadata‐based models have identified traditional CAD risk factors such as TC, LDL‐C, and age as major contributors. However, these factors alone could not account for CAD events observed in patients without such classic risk profiles [59]. In contrast, the superior performance of metagenomic and metabolomic models highlights the potential importance of gut microbial and metabolic alterations in the pathogenesis and progression of CAD [60].

Our study has several limitations. First, although our findings revealed strong associations and suggested potential links between specific microbes, metabolites, and ACS by including unique disease recovery group, some causal relationships remain to be validated and should be addressed in future studies using experimental approaches, such as through fecal microbiota transplantation and metabolite supplementation, in both preclinical and clinical settings. Second, the sample size is relatively modest, and participants were recruited from a single center in northern China. This may limit the generalizability of our findings to populations with different ethnic backgrounds, geographic locations, or lifestyles. Future studies with larger, multi‐center cohorts are needed to validate our results and to explore potential differences across ACS subtypes. Third, the cross‐sectional design precludes assessment of temporal dynamics or causality. Future longitudinal studies or randomized controlled trials are needed to validate these findings and further elucidate the directionality of the observed associations. Fourth, lifestyle‐related factors such as diet and physical activity were considered based on baseline data. In future studies, incorporating standardized tools such as food frequency questionnaires or 24‐h dietary recalls could enable more precise adjustment for potential confounders. Finally, while the term sCAD has been updated in the 2019 European Society of Cardiology (ESC) guidelines to chronic (or stable) coronary syndromes (CCS) [61], we have retained the original terminology in this study to remain consistent with the enrollment criteria and diagnostic definitions used at the time of participant recruitment.

Collectively, our study demonstrates that alterations in the gut microbiome, characterized by the enrichment of pro‐inflammatory taxa and functional metabolic shifts, are closely associated with CAD progression and the onset of ACS. By integrating metagenomic and metabolomic profiling, we identified robust microbial and metabolic signatures that distinguish CAD subtypes, including ACS‐specific features. The observed restoration of these ACS‐specific signatures in recovery sCAD patients suggests their potential involvement in disease resolution. These findings not only enhance our understanding of CAD pathogenesis but also provide candidate targets for stabilizing vulnerable plaques and modulating inflammation. Targeting gut microbial dysbiosis and associated metabolic pathways may offer novel strategies for ACS prevention and improved long‐term cardiovascular outcomes. Future studies should aim to validate these biomarkers in larger, diverse cohorts and assess the efficacy of microbiota‐modulating interventions in modifying disease risk.

## METHODS

### Participants and study design

Between March 2018 and December 2018, we continuously recruited 600 participants for whom there is complete information on medical history and clinical and biochemical parameters from Fuwai Hospital, National Center for Cardiovascular Diseases of China. Participants were divided into three groups based on clinical symptoms, signs, laboratory tests, electrocardiogram (ECG), CTA, and coronary angiographic results, which showed extent of arterial blockage and myocardial injury. The NCA group (*N* = 175) is comprised of healthy subjects or suspected patients with neither visible lesions or stenosis in coronary arteries shown on CTA or angiography, nor CAD‐related symptoms and signs. In the primary sCAD group (*N* = 161), patients were eligible if coronary angiography showed ≥50% of the luminal diameter of at least one native coronary vessel and they had no history of ACS before enrollment [62]. For our validation analysis, we also included an independent recovery sCAD group (*N* = 52) that consists of patients who had experienced an ACS event at least 3 months prior and had recovered to sCAD through revascularization and medications. The patients in the ACS group (*N* = 212) were clinically diagnosed with one of the following cardiovascular events: UAP, STEMI, or NSTEMI. UAP was diagnosed as a normal measurement of cardiac troponin (cTnI) and with at least one of the following criteria: prolonged (>20 min) angina pain at rest, new onset angina (Class II or III according to the Classification of the Canadian Cardiovascular Society), recent destabilization of previously stable angina with at least Canadian Cardiovascular Society Class III angina characteristics (crescendo angina), or post‐MI angina [2]. MI, including STEMI and NSTEMI, was diagnosed as a rise and/or fall of cardiac troponin with at least one value above the 99th percentile upper reference limit and with at least one of the following: (1) prolonged ischemia symptoms (>20 min), (2) new or presumed new significant ST‐segment‐T wave changes or new left bundle branch block, (3) development of pathological Q waves in the electrocardiogram, (4) imaging evidence of new viable myocardium loss or new regional wall motion abnormality, and (5) identification of an intracoronary thrombus by angiography or autopsy [63]. Patients who had one of the following criteria were excluded: (1) type 2, type 3, type 4a, type 4b, or type 5 universal classification of MI, (2) severe heart failure or cardiogenic shock (Killip grade more than II or NYHA class more than II), (3) mechanical complications of MI, (4) cardiac arrest or cardiopulmonary resuscitation after MI, (5) administration of antibiotics for more than 1 week in the previous 3 months [64], (6) a history of ACS or administration of coronary revascularization (including percutaneous coronary intervention and coronary artery bypass grafting) in the previous 3 months, (7) traumas or surgeries in the previous 3 months, (8) cerebrovascular diseases (including cerebral infarction and cerebral hemorrhage) in the previous 3 months, (9) gastrointestinal bleeding in the previous 3 months, (10) infectious diseases in the previous 3 months, (11) chronic gastrointestinal disease, (12) tumors, (13) autoimmune disorders, (14) renal dysfunction (severe renal disease creatinine >3.0 mg/dL) or a kidney transplantation history, and (15) consumption of probiotics, prebiotics, or yogurt within the past 7 days or habitual consumption. The angiographic data were confirmed independently by two observers in this study. The study was conducted according to the Principles of Helsinki Declaration and was approved by the Ethics Committee of Fuwai Hospital (approval number: 2018‐995). Written informed consents were obtained from all participants in this study.

### Collection and analysis of metadata

Via face‐to‐face questionnaire interviews, we collected metadata that covered participants' anthropometric features and information related to their health status, disease history, family history, medication use, physical activity, and dietary and lifestyle habits. Participant BMI was calculated as weight (kg) divided by squared height (m^2^). Waist circumference was measured by circling the abdomen in a horizontal direction 5 cm above the umbilicus. Smoking was defined as smoking at least one cigarette per day for over 1 year. Alcohol consumption was defined as at least 20 g/day for men and 10 g/day for women for over a year.

Peripheral blood samples for clinical chemistry analyses were taken after an overnight fast of at least 12 h into EDTA‐containing tubes and then tested as quickly as possible in laboratory equipment. Concentrations of plasma TC, TG, HDL‐C, LDL‐C, ApoA, ApoB, and FBG were measured using enzymatic techniques on a HITACHI 7150 autoanalyzer (Hitachi, Ltd.). TC, TG, HDL‐C, and LDL‐C concentrations were measured by enzymatic assay. ApoA and ApoB concentrations were measured by turbidimetric immunoassay. For individuals with triglyceride levels ≥400 mg/dL, LDL‐C was calculated indirectly using the Friedewald formula. LDL‐C was tested directly for individuals with triglyceride levels <400 mg/dL; hs‐CRP levels were determined using immunoturbidometry (Beckmann Assay 360). Plasma NT‐proBNP was measured using a commercial sandwich enzyme immunoassay (BI‐20852W, Biomedica). The normal range for plasma NT‐proBNP in our laboratory was <400 pmol/L. The concentration of serum Lp (a) was measured with an immuneturbidometry method (LASAY Lp (a) auto, SHIMA laboratories). The measurement range of Lp (a) levels was 5–1000 mg/L, and the normal limits were 0–300 mg/L. Scr was measured by an UniCelDxC 800 SYNCHRON System (Beckman Coulter), and the concentration was assessed using the Jaffe method. All other biomarkers were analyzed by standard hematologic and biochemical tests. Fresh feces of each subject were collected the first morning after admission to the hospital. Fecal and plasma samples were collected within a 24‐h window after hospital admission and before treatment. All samples were frozen on dry ice within 30 min and stored in −80°C freezers before further analysis [64].

Continuous variables were presented as means ± standard deviations (SD) if normally distributed or as medians with interquartile ranges (IQR) otherwise. Between‐group differences for normally distributed continuous variables were assessed using one‐way analysis of variance (ANOVA), while the Kruskal–Wallis *H*‐test was used for non‐normally distributed variables. *χ* ^2^ test was applied in comparing categorical variables, and Fisher's exact test was applied when the expected counts were less than 5.

### Human fecal sample collection and DNA extraction

Fresh feces samples were collected and stored at −80°C until delivered from Fuwai Hospital to the laboratory in an ice bag using insulating polystyrene foam containers. DNA was extracted using an EZNA™ stool DNA isolation kit (Omega Bio‐Tek, VWR) according to the manufacturer's instruction [64].

### DNA library construction and sequencing

The DNA library was constructed using the TruSeq Nano DNA LT Library Preparation Kit (FC‐121‐4001, Illumina). DNA was fragmented by dsDNA Fragmentize (M0348S, NEB) via incubation at 37°C for 30 min. Fragments were blunt‐ended using a combination of fill‐in reactions and exonuclease activity, and adapters were ligated to the fragments. The ligated products were amplified with PCR at LC‐Bio Technology Co., Ltd. with the following conditions: initial denaturation at 95°C for 3 min; 8 cycles of denaturation at 98°C for 15 s, annealing at 60°C for 15 s, and extension at 72°C for 30 s; final extension at 72°C for 5 min. The resulting libraries were sequenced on an Illumina HiSeq. 4000 sequencer (Illumina). The running mode of metagenomics was paired‐end of 150 bp [64].

### Sequencing data analysis

Quality control (QC) for the metagenomics shotgun sequencing data was conducted using FastQC (http://www.bioinformatics.babraham.ac.uk/projects/fastqc/). Low‐quality reads and adapter sequences were removed with Trimmomatic. The reads that could be aligned with the human genome were cleaned by using SOAP aligner software (v2.21). Taxonomic profiles were generated using MetaPhlAn4 [65]. Alpha diversity indices, including richness and evenness, were calculated using R package vegan package. The principal coordinate analysis (PCoA) was calculated based on the Bray–Curtis distance using vegan. Gene family (KEGG ontology) and pathway profiling were done using HUMAnN3, which provides microbial gene family (90% similarity) quantifications that are further stratified by contributing organisms [66]. The relationships between pathways and metabolites were analyzed using the MetaCyc database [67].

### Plasma sample collection and preparation for metabolomics analysis

Fasting plasma samples were collected in heparinized tubes, chilled to 4°C, centrifuged within 1 h of collection (12 min at 1350 rpm), separated into aliquots, and stored at −80°C. The same treatment was performed as follows [68]. One hundred microlitres of plasma sample and QC sample was transferred into a 10 mL glass tube and 20 μL internal standards (including 3 μg/mL of DL‐valine‐d8; 1 μg/mL of L‐tryptophan‐d5 (indole‐d5); 5 μg/mL of succinic acid‐2,2,3,3‐d4; 1 μg/mL of TG (16: 0/18: 0/16:0‐d5); PC (16: 0‐d31/18:1) and 2 μg/mL of oleic acid‐d9). Three milliliters of methanol/methyl tert‐butyl ether (1:1, v/v) was added to the tube, and the sample was vortexed for 5 min at 2500 rpm and then centrifuged at 4200 rpm for 10 min (4°C). The solution, other than the protein precipitate, was transferred to another glass tube. 3.0 mL of methyl tertbutyl ether and 1.2 mL of pure water were then added, and the tube was vortexed at 2500 rpm for 15 min and then centrifuged at 4200 rpm for 10 min (4°C). The organic supernatant was transferred into another glass tube. Both the organic phase and aqueous phase were dried under a gentle nitrogen stream. The aqueous residue was re‐dissolved in 100 μL acetonitrile/H_2_O (2:98, v/v) for metabolomics analysis. The organic residue was re‐dissolved in 400 μL methanol/chloroform (1:1, v/v) for lipidomic analysis. To monitor data quality and instrument stability, QC samples containing aliquots of plasma samples from all participating subjects were processed in parallel, and a QC sample was inserted every 10 samples throughout the sequences. The order of sample preparation and injection was randomized to avoid system biases [69].

### UPLC‐MS experiment parameters for metabolomics and lipidomics analysis

Plasma samples were assigned to untargeted or targeted metabolomics analysis based on the order of enrollment. To discover the potential differential metabolites, global metabolic profiling was performed on a training set of 287 human plasma samples (91 NCA, 78 primary sCAD, 102 ACS, and 16 recovery sCAD, 13 samples not available to be measured) using UPLC‐HRMS. A two‐dimensional ultrahigh‐performance liquid chromatography system (ACQUITY UPLC I‐Class, Waters) was used for the separation on a Waters HSS T3 column (1.8 μm, 100 mm × 2.1 mm) at a column temperature of 35°C (polar metabolites)/45°C (lipid metabolites). The flow rate was 0.25 mL/min. The injection volume was 10 μL/5 μL. For the polar extracts, mobile phase A was water containing 0.1% formic acid and mobile phase B was acetonitrile. The gradient was programmed as follows: 0–9 min, 2%–60% B; 9–18 min, 60% B; 18–20 min, 60%–100% B; 20–30 min, 100% B. For the lipid extracts, mobile phase A was comprised of 2 mM ammonium acetate in water containing 0.1% formic acid, mobile phase B was comprised of acetonitrile/isopropanol (50:50, v/v) containing 2 mM ammonium acetate and 0.1% formic acid. The gradient was programmed as follows: 0–1 min, 35%–50% B; 1–3 min, 50%–70% B; 3–10 min, 70%–90% B; 10–15 min, 90%–100% B; 15–30 min, 100% B. The mass spectrometric data were collected using a Q‐Orbitrap MS instrument (Q‐Exactive, Thermo Fisher Scientific) equipped with an electrospray ionization (ESI) source. The parameters for the MS instrument in positive/negative ion mode were sheath gas 40/45 arb; spray voltage, 3.5/−3.5 kV; capillary temperature, 350°C; resolution, 70,000; automatic gain control target, 3e6; and maximum injection time of 100 ms. The scan range was *m/z* 70–1000 (polar metabolites)/80–1200 (lipid metabolites).

Another 285 human plasma samples (73 NCA, 77 primary sCAD, 106 ACS, and 29 recovery sCAD, 15 samples not available to be measured) were used for targeted analysis as a test set to quantify differential metabolites. Trimethylamine‐d9 N‐Oxide, phenylacetyl‐d5 L‐glutamine, L‐lysine‐4,4,5,5‐d4 hydrochloride, D‐phenylalanine‐d5, indole‐2,4,5,6,7‐D5‐3‐acetate, L‐tyrosine‐^13^C9,^15^N, choline‐d9 chloride; succinate‐2,2,3,3‐d4, and L‐tryptophan‐d5 were chosen as the internal standards to monitor the stability of the analysis and saved as a quantitative calibration factor [70]. The quantification analysis was performed on the same Waters ACQUITY UPLC system coupled to a 5500 Q‐Trap system (AB SCIEX) with an ESI in scheduled MRM mode. The separation was conducted on a Waters HSS T3 column (1.8 μm, 100 mm × 2.1 mm) at a column temperature of 35°C, and the mobile phase was water containing 0.1% formic acid (A) and acetonitrile (B), at a flow rate of 0.25 mL/min for both positive and negative ionization modes. An optimized elution gradient of 2% B → 100%B in 0–10 min; 100% B for 10–11 min; and 2% B for 11.1–16 min was used. The declustering potentials, collision energies, and suitable product ions for each variable were optimized to obtain the best signals ^77^. Other MS parameters were set as: gas1, 50 psi; gas2, 50 psi; curtain gas, 20 psi; source temperature, 450°C; and ion spray voltage, 5500/−4500 V.

### Data processing for metabolomics and lipidomics analysis

The UPLC‐MS raw/wiff data files were processed with the bioinformatics software Progenesis QI (Waters), which includes alignment reference selection, alignment, normalization, matching, and identification. A data matrix consisting of the retention time, *m/z* value, and peak area was obtained. The data of polar extracts were analyzed by using the established “Metabolic Pathway Mapping” data processing strategy based on mummichog. For the lipid extracts, the normalized preprocessed data sets were imported into the SIMCA‐P 14.0 software package (Umetrics AB, Ume°a) for orthogonal partial least‐squares discriminant analysis and permutation tests. To identify reliable differential variables, only those with a variable importance for the projection (VIP) >1.5 were taken into consideration; adduct and isotope ions, as well as variables with crossover between groups, were deleted. For both polar and lipid extracts, differential metabolite ions were identified with MaAsLin2 (*q* < 0.1; see *Confounding factors analysis* section for details on adjustment for covariates). Differential metabolites were identified according to their exact *m/z* values, MS/MS spectra, and retention times. Following the results of the above differential metabolites analysis, the differential metabolites were further used for the KEGG pathway enrichment analysis via MetaboAnalyst (https://www.metaboanalyst.ca/). Metlin (https://metlin.scripps.edu/), HMDB (https://hmdb.ca/), ALEX123 lipid calculator (http://alex123.info/ALEX123/MS.php), and LIPID MAPS (http://www.lipidmaps.org/) were used for database searching. MultiQuant software (version 3.0.3, AB SCIEX) was used for targeted quantitative analysis.

### Confounding factors analysis

We systematically evaluated the effect of clinical variables on our analysis, including age, sex, smoking history, BMI, and medication usage [14]. The medications assessed encompassed antiplatelet agents (aspirin, clopidogrel, ticagrelor), lipid‐lowering agents (statins), anti‐hypertensive drugs (ACE inhibitors or ARBs [angiotensin‐converting enzyme inhibitors or angiotensin receptor blockers], beta‐blockers, calcium channel blockers, diuretics), and antidiabetic medications (insulin, metformin). We employed PERMANOVA to discern the effects of these confounding factors on both microbial community composition and metabolite profiles. By comparing the statistical significance of between‐group (PERMANOVA *p*‐value) and within‐group (beta dispersion *p* value) differences, we were able to determine whether observed variations were attributable to biological differences or to the influence of confounding variables. We employed the statistical tool MaAsLin2 to correct for confounding factors and identify the gut microbiota and metabolites most closely associated with disease status [71].

### Mediation analysis methods

We performed two mediation analyses: (1) assessing whether the association between gut microbes and metabolite levels was mediated through specific microbial pathways, and (2) assessing whether the association between gut microbes and ACS was mediated through key metabolites [72]. Microbial abundances, pathway abundances, and metabolite levels were log‐transformed and z‐score standardized. The mediation R package was used to estimate the average causal mediation effect, average direct effect, total effect, and proportion mediated, adjusting for relevant covariates.

### Identification of markers to distinguish different CAD stages using machine learning models

To identify markers capable of differentiating various stages of CAD, we constructed classification models (NCA, sCAD, ACS) using the LightGBM algorithm. The models integrated several data sources: clinical laboratory data, targeted quantified metabolites, and microbiome profiles (species‐level relative abundances obtained with MetaPhlAn4). The data set comprised 285 samples with corresponding targeted metabolomics quantification. A stratified 80% subset was used as a discovery cohort, further classified into training and validation sets for 10‐fold cross‐validation. The remaining 20% subset was reserved as an independent hold‐out data set for validation and utilized as test data to ensure model robustness and generalizability.

Before model training, all features underwent selection via the Boruta algorithm implemented with a RandomForestClassifier backbone (n_estimators = ‘auto’, max_depth = 5, class_weight = ‘balanced’, random_state = 1). This algorithm, an extension of the random forest methodology, systematically excises features deemed less relevant than random probes, as determined by two‐tailed statistical testing with Bonferroni correction (alpha = 0.05, max_iter = 100, perc = 90) [73]. GridSearchCV [74], a hyperparameter tuning method provided by the scikit‐learn library, was used to conduct hyperparameter optimization through an exhaustive search over a predefined parameter grid. Subsequently, the classification models were constructed utilizing the LightGBM algorithm within the gradient boosting decision tree domain [75].

The model was ultimately applied to the independent hold‐out data set for validation. All analytical procedures were executed utilizing the Python “scikit‐learn” package. The contributions of different features to the models were quantified and presented as feature importances. Model performance was evaluated by computing the area under the receiver operating characteristics curve (ROC‐AUC). The SHapley Additive exPlanations (SHAP) framework was employed to visually elucidate which features contributed the most to the integrated model. SHAP summary plots were generated to visualize feature importance with matplotlib, depicting each sample as a dot colored by feature value (red: high, blue: low) and sized by sample weight. The color intensity of each feature indicates both the direction and extent of its contribution to the model's predictive output.

### Statistical analysis

Analyses were carried out using R (version 4.4.2, https://www.R-project.org/), or with Python 3.9. The pairwise correlation coefficients in Figure 4C were calculated using Spearman's rank correlation coefficients. For univariate analysis across groups, overall differences were first assessed using the Kruskal–Wallis test, followed by post hoc Dunn's tests for pairwise comparisons with Benjamini–Hochberg (BH) adjusted *p*‐values. MetOrigin (https://metorigin.met-bioinformatics.cn/home/) was applied to explore the links among species, metabolome, and pathways [37]. Fisher's exact test was employed for group comparisons of clinical indicators related to diet, exercise, and sleep. *p* < 0.05 was considered statistically significant.

## AUTHOR CONTRIBUTIONS

**Jing Xu**: Conceptualization; methodology; software; validation; formal analysis; data curation; writing—original draft; writing—review & editing; visualization; project administration. **Die Dai**: Methodology; software; validation; formal analysis; data curation; writing—original draft; writing—review and editing; visualization. **Yanan Yang**: Methodology; software; validation; formal analysis; data curation; writing—original draft; visualization. **Shanshan Gao**: Conceptualization; methodology; validation. **Jingang Yang**: Investigation; resources. **Chaoran Dong**: Investigation; resources. **Weixian Yang**: Funding acquisition; investigation; resources. **Jiansong Yuan**: Investigation; resources. **Tianjie Wang**: Investigation; resources. **Tao Tian**: Investigation; resources. **Yanmin Yang**: Investigation; resources. **Fang Luo**: Investigation; resources. **Ping Jiang**: Investigation; resources. **Chao Wu**: Resources; investigation. **Xiaolu Sun**: Investigation; resources. **Yonggang Sui**: Resources; investigation. **Guofeng Gao**: Investigation; resources. **Wentao Ma**: Resources; investigation. **Yuan Wu**: Investigation; resources. **Jun Zhang**: Resources; investigation. **Jia Li**: Investigation; resources. **Chao Guo**: Resources; investigation. **Cheng Cui**: Investigation; resources. **Tingting Guo**: Investigation; resources. **Xueyan Zhao**: Resources; investigation. **Jinqing Yuan**: Investigation; resources. **Shubin Qiao**: Resources; investigation. **Fenghuan Hu**: Investigation; resources. **Xiaojin Gao**: Investigation; resources. **Xiaoliang Luo**: Resources; investigation. **Haoran Peng**: Writing—review & editing. **Daoming Wang**: Writing—review & editing. **Jiqiu Wu**: Writing—review & editing. **Chongming Wu**: Conceptualization; validation; Writing—review & editing; supervision; project administration. **Jiuming He**: Conceptualization; methodology; validation. **Wei‐Hua Chen**: Conceptualization; project administration; supervision; validation; writing—review & editing. **Yuejin Yang**: Writing—review & editing; project administration; funding acquisition; conceptualization; supervision; validation. **Jingyuan Fu**: Conceptualization; project administration; supervision; validation; writing—review & editing.

## CONFLICT OF INTEREST STATEMENT

The authors declare no conflicts of interest.

## ETHICS STATEMENT

The study was conducted according to the guidelines of the Helsinki Declaration and was approved by the Ethics Committee of Fuwai Hospital (No. 2018‐995). Written informed consents were obtained from all participants.

## Supporting information


**Figure S1:** Distribution of self‐reported lifestyle and dietary habit indicators across participant groups.
**Figure S2:** Abundances of pro‐inflammation bacteria (refer to literature, Table S2) within the NCA, primary sCAD, and ACS groups.
**Figure S3:** Volcano plots highlighting differential polar and lipid metabolites among the NCA, primary sCAD, and ACS groups.
**Figure S4:** Aromatic amino acid/TMAO metabolism‐related metabolites in ACS.
**Figure S5:** Mediation analysis linking gut microbes, microbial pathways, plasma metabolites, and ACS.
**Figure S6:** SHAP (SHapley Additive exPlanations) value summary plots to illustrate the contribution of each feature to the model's predictions.

**Table S1:** Baseline characteristics of included participants.
**Table S2:** List of the functional bacteria.
**Table S3:** Gut bacterial species progressively increased/decreased in CAD patients.
**Table S4:** Identified pathways of metagenomic data by HUMAnN3 analysis.
**Table S5:** Statistics of differential pathways between groups from metagenomic data.
**Table S6:** Significantly different polar metabolites identified by metabolomics.
**Table S7:** Significantly different lipids identified by metabolomics.
**Table S8:** ACS‐specific species identified by metagenomics.
**Table S9:** ACS‐specific metagenomic pathways identified by HUMAnN3 analysis.
**Table S10:** ACS‐specific metabolites identified by metabolomics.
**Table S11:** Baseline characteristics of primary sCAD and recovery sCAD patients.
**Table S12:** Results of targeted metabolomics.
**Table S13:** Summary of mediation analysis linking gut microbes, microbial pathways, plasma metabolites, and ACS.

## Data Availability

The data that support the findings of this study are available on request from the corresponding author. The data are not publicly available due to privacy or ethical restrictions. Metagenomic sequencing data associated with this study are deposited in the BioProject database of the National Genomics Data Center (NGDC, https://ngdc.cncb.ac.cn/bioproject/) under the accession number PRJCA017875 (https://ngdc.cncb.ac.cn/bioproject/browse/PRJCA017875) and are available under controlled access. Due to ethical and legal restrictions, participant‐level data are available for research purposes only upon request, subject to approval by the Ethics Committee of Fuwai Hospital and the execution of appropriate data transfer agreements. This includes submitting a research proposal to the corresponding author Yuejin Yang (yangyjfw@126.com), where upon approval, all data analysis need to be done on a local server with protected access, in compliance with Personal Information Protection Law of the People's Republic of China (PIPL) and Regulations on the Administration of Human Genetic Resources of the People's Republic of China. Computational analyses were performed using the bioBakery suite of tools; species‐level microbial abundances were computed using MetaPhlAn v.4.0 (https://github.com/biobakery/MetaPhlAn). Functional potential profiling was carried out with HUMAnN v.3.0 (https://github.com/biobakery/humann; Methods). The aggregated data and scripts used in the study are saved in Github (https://github.com/JingXu0701/CAD_MultiOmics). Supplementary materials (figures, tables, graphical abstract, slides, videos, Chinese translated version, and update materials) may be found in the online DOI or iMeta Science http://www.imeta.science/.
