# Supplementary material for: Distinct microbial and metabolic shifts characterize acute coronary syndrome and recovery
Source: Imeta. 2025 Sep 19;4(5):e70079. doi: 10.1002/imt2.70079 (PMC12528003; doi:10.1002/imt2.70079)
Supplement: Supplementary file 1 — Figure S1: Distribution of self‐reported lifestyle and dietary habit indicators across participant groups. Figure S2: Abundances of pro‐inflammation bacteria (refer to literature, Table S2) within the NCA, primary sCAD, and ACS groups. Figure S3: Volcano plots highlighting differential polar and lipid metabolites among the NCA, primary sCAD, and ACS groups. Figure S4: Aromatic amino acid/TMAO metabolism‐related metabolites in ACS. Figure S5: Mediation analysis linking gut microbes, microbial pathways, plasma metabolites, and ACS. Figure S6: SHAP (SHapley Additive exPlanations) value summary plots to illustrate the contribution of each feature to the model's predictions. [file IMT2-4-e70079-s001.docx]

**Supporting information to**

**Distinct microbial and metabolic shifts characterize acute coronary syndrome and recovery**

**Running title:** Gut microbiota and metabolite signatures in ACS

Jing Xu^1, 2#^, Die Dai^3#^, Yanan Yang^4#^, Shanshan Gao^5#^, Jingang Yang^1#^, Chaoran Dong^5^, Weixian Yang^1^, Jiansong Yuan^1^, Tianjie Wang^1^, Tao Tian^1^, Yanmin Yang^1^, Fang Luo^1^, Ping Jiang^1^, Chao Wu^1^, Xiaolu Sun^1^, Yonggang Sui^1^, Guofeng Gao^1^, Wentao Ma^1^, Yuan Wu^1^, Jun Zhang^1^, Jia Li^1^, Chao Guo^1^, Cheng Cui^1^, Tingting Guo^1^, Xueyan Zhao^1^, Jinqing Yuan^1^, Shubin Qiao^1^, Fenghuan Hu^1^, Xiaojin Gao^1^, Xiaoliang Luo^1^, Haoran Peng^2^, Daoming Wang^2^, Jiqiu Wu^2^, Chongming Wu^4*^, Jiuming He^5*^, Wei-Hua Chen^3,6*^, Yuejin Yang^1*^, Jingyuan Fu^2*^

^1^ Department of Cardiology, State Key Laboratory of Cardiovascular Diseases, Fuwai Hospital, National Center for Cardiovascular Diseases, Chinese Academy of Medical Sciences and Peking Union Medical College, Beijing 100037, China

^2^ Department of Genetics & Department of Pediatrics, University Medical Center Groningen, University of Groningen, Groningen 9700 RB, The Netherlands

^3^ Key Laboratory of Molecular Biophysics of the Ministry of Education, Hubei Key Laboratory of Bioinformatics and Molecular Imaging, Department of Bioinformatics and Systems Biology, Center for Artificial Intelligence Biology, College of Life Science and Technology, Huazhong University of Science and Technology, Wuhan 430074, China

^4^ School of Chinese Materia Medica, Tianjin University of Traditional Chinese Medicine, Tianjin 301617, China

^5^ State Key Laboratory of Bioactive Substance and Function of Natural Medicines, Institute of Materia Medica, Chinese Academy of Medical Sciences and Peking Union Medical College, Beijing 100050, China

^6^ School of Biological Science, Jining Medical University, Rizhao 276800, China

**^#^** These authors contributed equally to this work: Jing Xu, Die Dai, Yanan Yang, Shanshan Gao, Jingang Yang

* Correspondence: yangyjfw@126.com (Yuejin Yang), j.fu@umcg.nl (Jingyuan Fu), weihuachen@hust.edu.cn (Wei-Hua Chen), hejiuming@imm.ac.cn (Jiuming He), chomingwu@163.com (Chongming Wu).

**Supplementary figures**

**
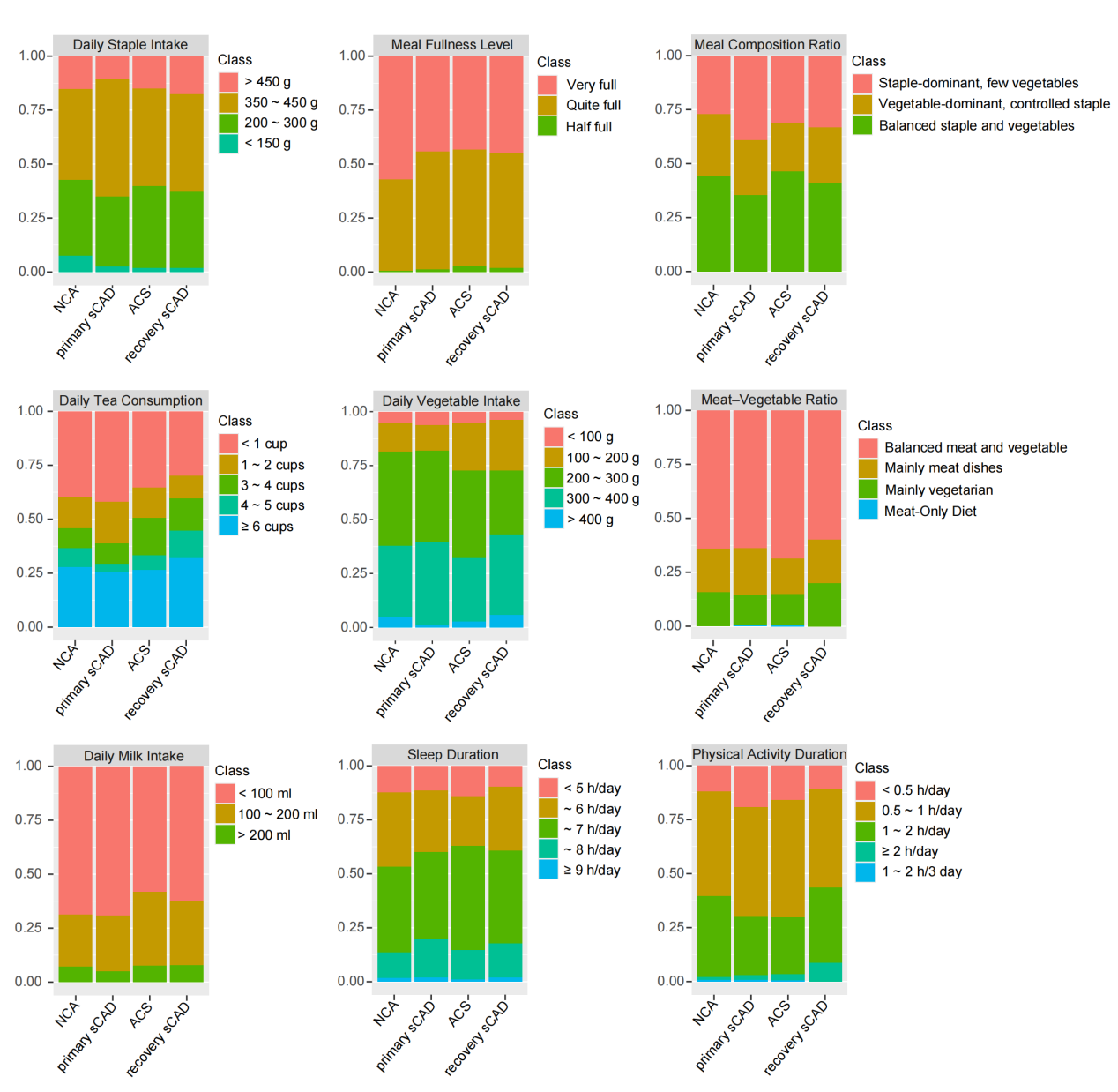
**

**Figure S1 Distribution of self-reported lifestyle and dietary habit indicators across participant groups**. Variables include meal fullness, staple–vegetable ratio, meat–vegetable balance, daily intake of staple foods, vegetables, tea, and milk, as well as sleep duration and physical activity time. No significant differences were observed across groups (Fisher’s exact test, all *p* > 0.05).


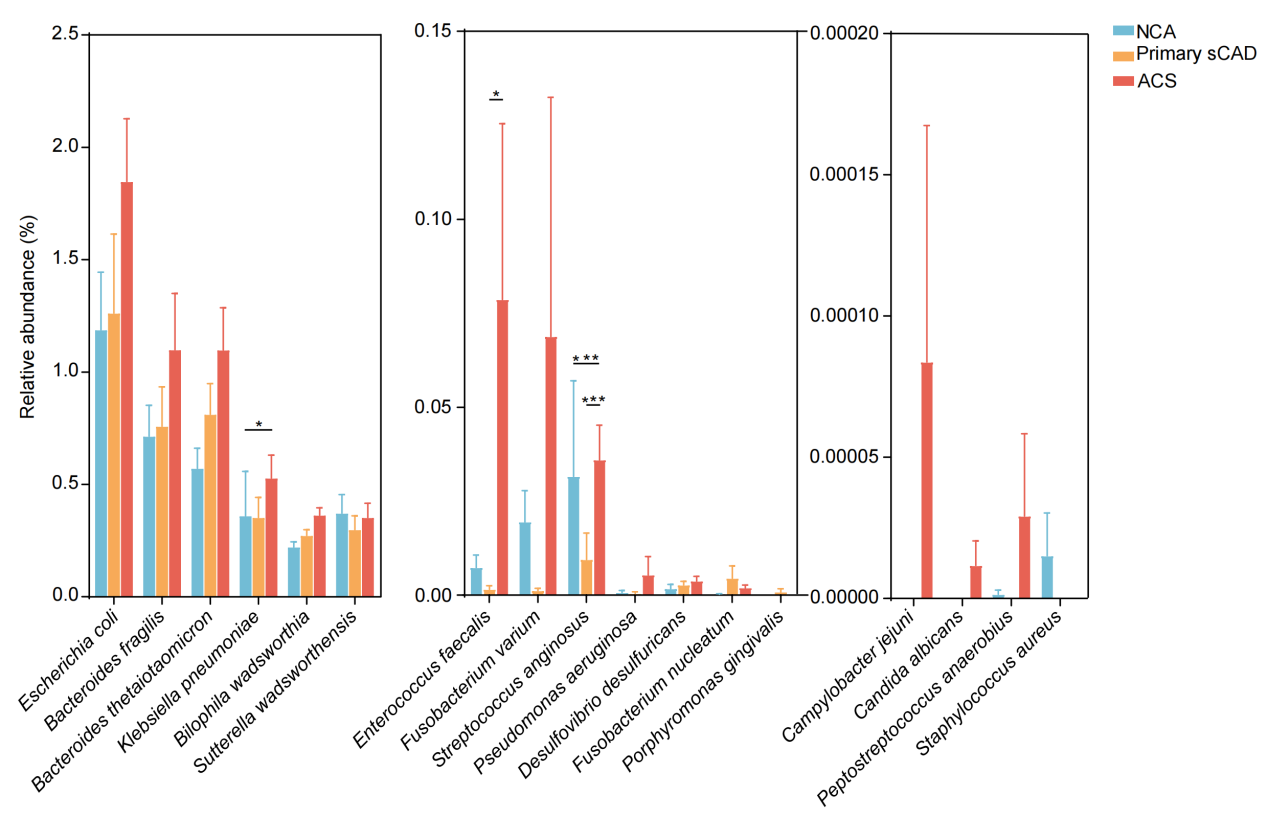
**Figure S2** **Abundances of pro-inflammation bacteria (refer to literature, Table S2) within the NCA, primary sCAD, and ACS groups.** Significance levels based on *q*-values from the MaAsLin2 analysis: * *q* < 0.1, ** *q* < 0.01, and *** *q* < 0.001.


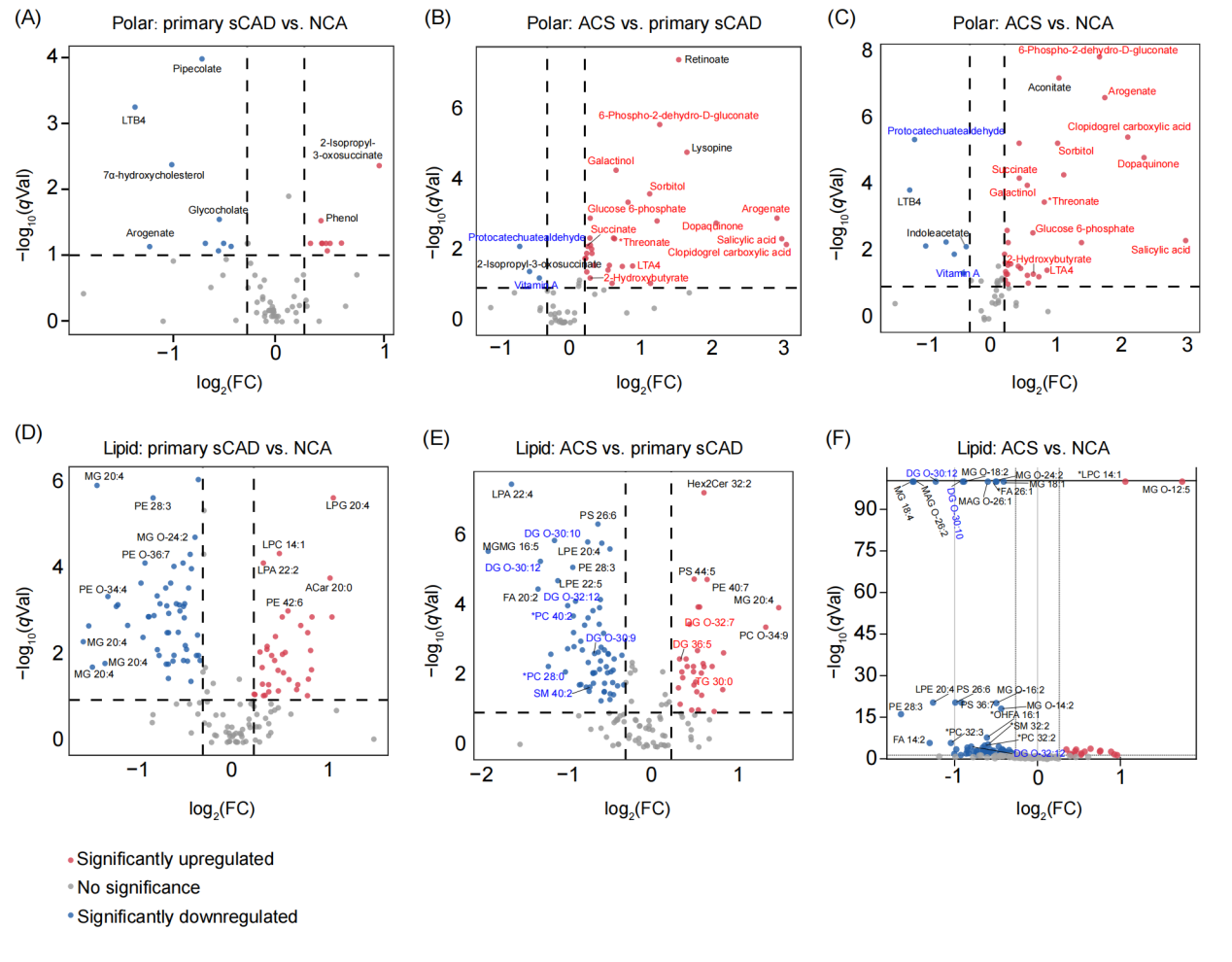


**Figure S3 Volcano plots highlighting differential polar and lipid metabolites among the NCA, primary sCAD, and ACS groups.** (A−C) Polar metabolites: (A) primary sCAD vs. NCA, (B) ACS vs. primary sCAD, and (C) ACS vs. NCA. (D−F) Lipid metabolites: (D) primary sCAD vs. NCA, (E) ACS vs. primary sCAD, and (F) ACS vs. NCA. Significance levels are based on *q*-values from the MaAsLin2 analysis.


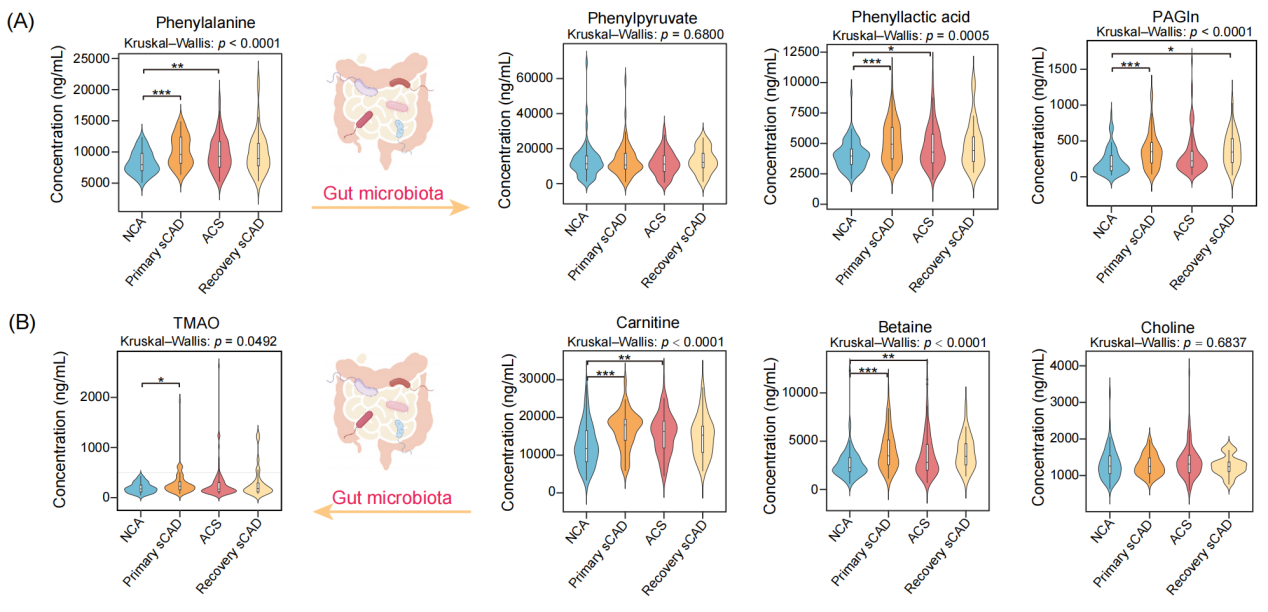


**Figure S4 Aromatic amino acid/TMAO metabolism-related metabolites in ACS.** (A) Concentrations of phenylalanine, phenylpyruvate, phenyllactic acid, and PAGIn. (B) Concentrations of TMAO, carnitine, betaine, and choline. *p*-values were computed using the Kruskal-Wallis H-test and the post-hoc Dunn’s test. Asterisks denote significance levels: * *p* < 0.05, ** *p* < 0.01, and *** *p* < 0.001.

**
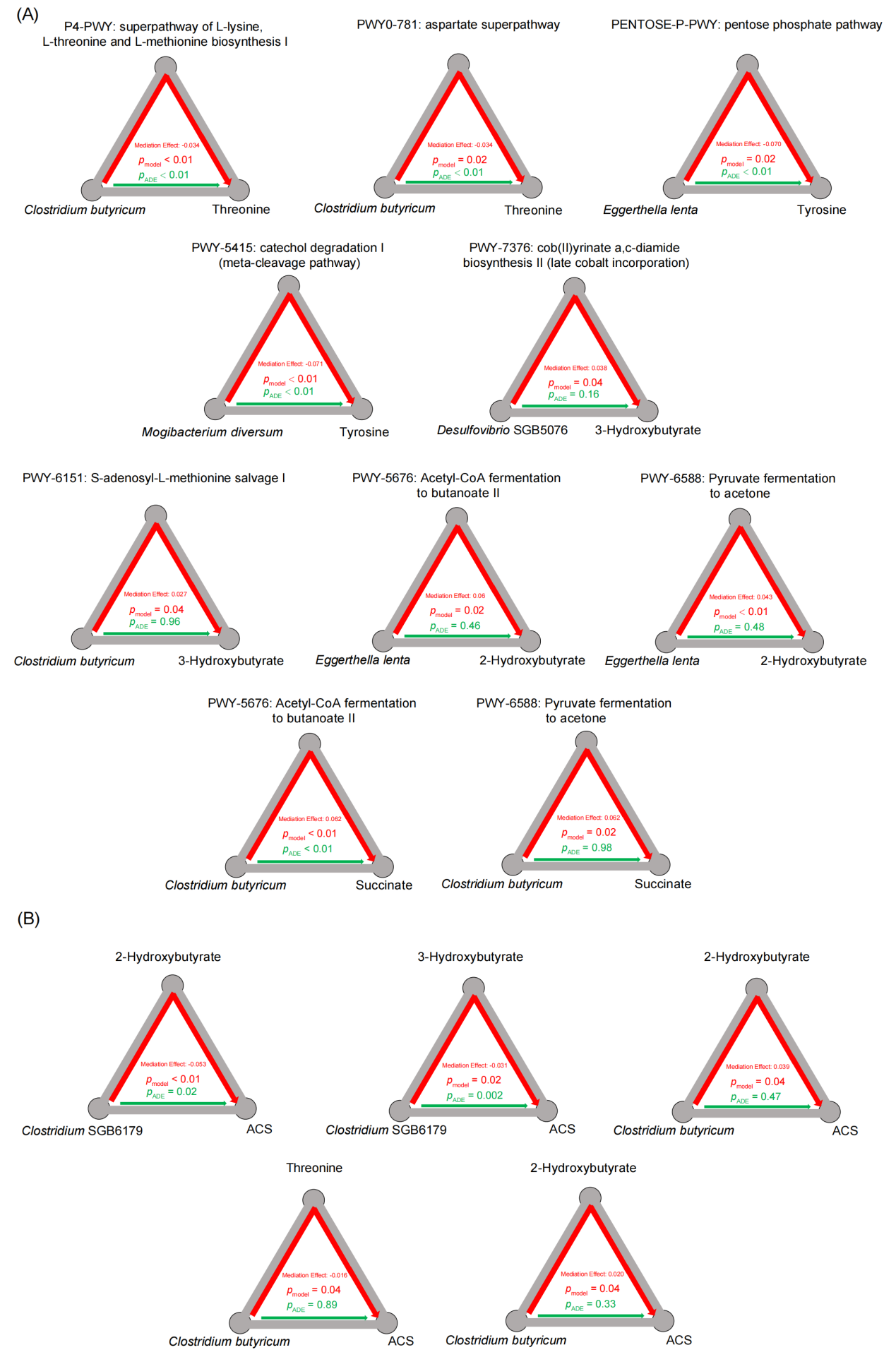
**

**Figure S5 Mediation analysis linking gut microbes, microbial pathways, plasma metabolites, and ACS.** (A) Mediation analysis assessing whether the associations between gut microbes and key metabolites were mediated through specific microbial functional pathways. (B) Mediation analysis assessing whether the associations between gut microbes and ACS were mediated through key metabolites. Microbial abundances, pathway abundances, and metabolite levels were log-transformed and z-score standardized prior to analysis. Red lines indicate the mediation path, with corresponding mediation effect sizes shown. p values are reported for the mediation model (p _model_) and the average direct effect (p _ADE_).


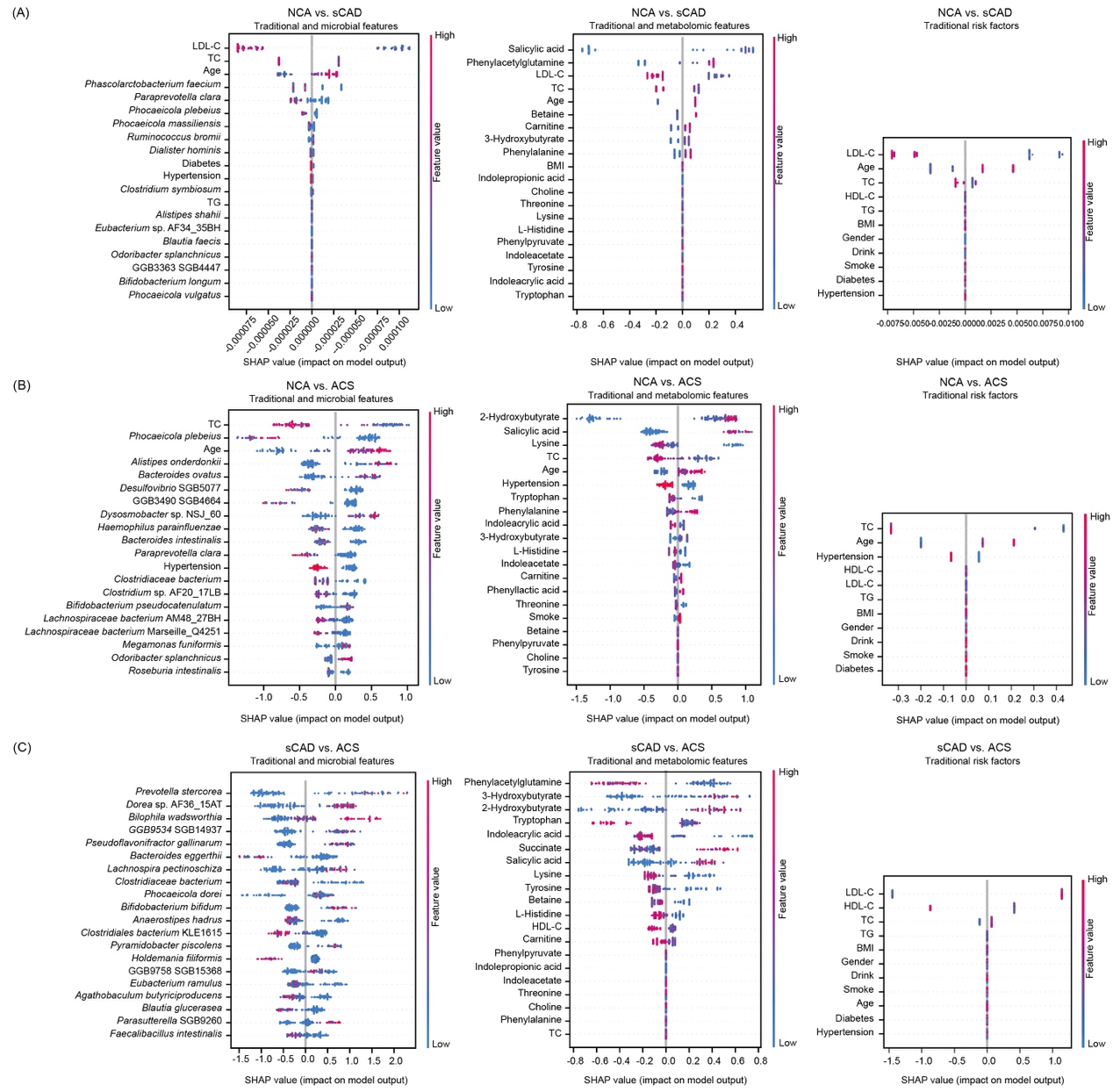


**Figure S6 SHAP (SHapley Additive exPlanations) value summary plots to illustrate the contribution of each feature to the model's predictions.** (A-C) SHAP plots for differentiating (A) NCA and sCAD, (B) NCA and ACS, and (C) sCAD and ACS based on traditional and microbial features, traditional and metabolomic features, and traditional risk features. Main features are ranked according to their importance, as determined by the absolute value of their SHAP values. Plots display features along the x-axis, with their corresponding SHAP values on the y-axis. Clinical indicators, microbial species, and metabolite levels are represented as distinct categories, and the color intensity of each feature indicates the direction and magnitude of its contribution to the model's output.
